# Thermomechanical biorefining of *Pinus radiata* biomass to produce biochemicals using reactive extrusion

**DOI:** 10.1186/s40643-025-00971-9

**Published:** 2025-11-05

**Authors:** Beatrix Theobald, Aaron Tay, Sumanth Ranganathan, Queenie Tanjay, Sunita Patel, Rebecca van Leeuwen, Marc Gaugler

**Affiliations:** Scion Group, Bioeconomy Science Institute, Titokorangi Drive, Private Bag 3020, 3046 Rotorua, New Zealand

**Keywords:** Reactive extrusion, Biochemicals, Biorefinery, Green manufacturing, Lignocellulosic biomass

## Abstract

Currently, ca. 30 million m^3^ of *Pinus radiata* are harvested annually in New Zealand to produce timber, pulp and paper, with by-products such as bark and sawdust generated during processing. The most common use for sawdust is as a solid fuel for process heat. However, it is a feedstock that can be processed into platform biochemicals. Although conversion processes focusing on biochemical production from wood are scarce, they are becoming more commercially established. Here, reactive extrusion was explored as a continuous, fast method to depolymerise sawdust into soluble biochemicals with residence times of less than two minutes. This is substantially shorter than other biotechnology routes or conventional batch processing and highlights the potential for integration of reactive extrusion into biorefinery operations. While conventional wood extrusion focused on the solid fraction, this work extensively investigated the liquid biochemical profile. The effects of temperature, moisture content, screw speed, and screw design on the biochemical yield from sawdust were studied. The results indicated that kneading elements in the screw design were key to achieving good processing of the sawdust. A high moisture content of 50% (by weight) was instrumental in the isolation of biochemicals. Moreover, the screw speed had little to no effect on the biochemical composition obtained from the reactive extrusion process. Finally, a maximum of 6.5–7.5% of biochemicals were recovered from sawdust in the liquid phase when processed between 325 °C and 375 °C. The biochemical analyses of the liquor showed a high amount of acetic acid (up to 7913 mg/L) and methanol (up to 2277 mg/L). Furthermore, the furanic content increased with an increase in temperature between 275 °C and 375 °C, while an inverse trend was observed for aromatic phenols. The analyses also revealed that lignin and hemicellulose were depolymerised to produce oligomeric and monomeric breakdown products, while cellulose was untouched. This study successfully demonstrated the successful use of a twin-screw reactive extruder to continuously produce a biochemical-rich liquor from sawdust.

**Graphical abstract:**

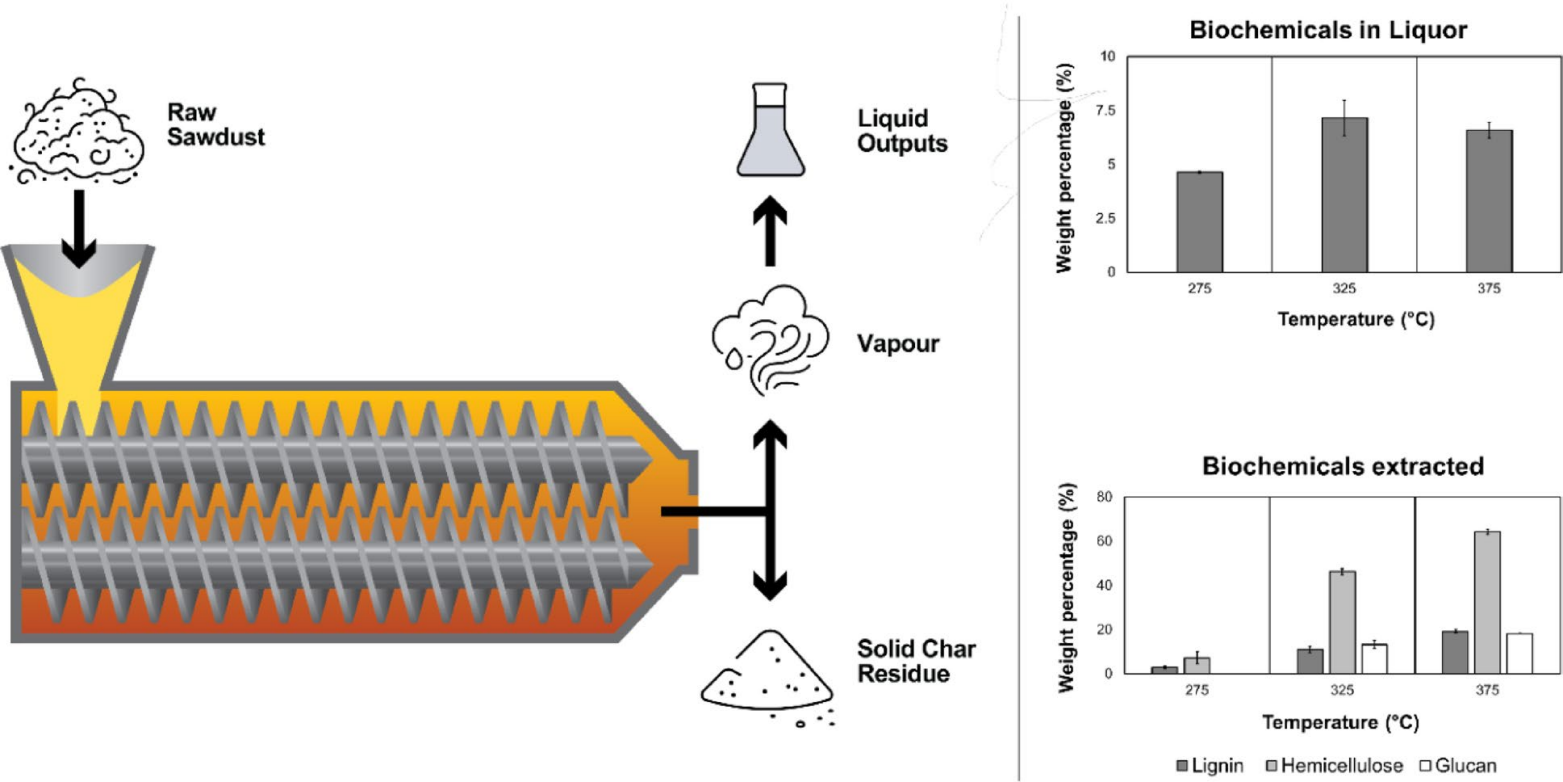

**Supplementary Information:**

The online version contains supplementary material available at 10.1186/s40643-025-00971-9.

## Introduction

Renewable resources and materials are becoming increasingly important and sought after due to current economic, environmental, and geopolitical factors. There is a need to reduce greenhouse gas emissions, and one such strategy is to shift from fossil fuel-derived products to renewable sources (Lamb et al. 2021). Countries and industries have recognised that greater energy, resource self-sufficiency and stable supply chains are possible by investing in local and sustainable resources. Consequently, governments worldwide are implementing policies and incentives to promote sustainable and renewable resource options, for example, the European Union's Green Deal and China's 14th Five‐Year Plan, which include steps to promote the bioeconomy. At the same time, advances in science, research, and technology related to bio-manufacturing, biotechnology, and bio-based materials continue to create new opportunities.

One such technology area is biorefining, the synergistic process of converting biomass into various chemicals, materials, and energy (Berntsson et al. 2012; Stafford et al. 2020). The concept has been derived from traditional petroleum refineries, whereby crude oil is replaced with available biobased materials, for example, wood (Liao et al. 2020), straw (He et al. 2023), algae (Koyande et al. 2019), or other solid or liquid primary industry by-products (Srivastava et al. 2023; Venkata Mohan et al. 2016). The global landscape of biorefineries has been evolving, with various reports indicating changes in their number and capacity. According to the IEA Bioenergy's "Global Biorefinery Status Report 2022", there were 1312 operational biorefineries worldwide at the time (IEA-Bioenergy, 2022b), and a continued expansion of biorefinery operations is expected (BCC-Research 2024; Research-and-Markets 2024). The most utilised primary biomass feedstocks for biorefinery are starch or oil crops. Despite being the most abundant organic resource on Earth (Mathew & Zakaria 2015; Singh et al. 2022), along with being readily available, renewable, and sustainable, lignocellulosic biomass (LCB) is only the third most used primary biomass feedstock. Even when considering secondary biomass, i.e. residues from agriculture and forestry, LCB utilisation remains relatively low, with use in only at least 24% of all biorefinery operations worldwide in 2022. There are opportunities for growth in this area. In particular, wood has emerged as a critical LCB source (Casau et al. 2022; Stafford et al. 2020). Estimates suggest that, as of 2020, 4.3 billion m^3^ of wood is processed annually worldwide (Wijeyekoon & Vaidya 2021), representing a large potential feedstock for biorefinery purposes (Braghiroli & Passarini 2020; Sulis et al. 2025).

Woody LCB consists of three biopolymers: lignin (10–25%), cellulose (40–60%), and hemicellulose (15–30%), along with minor amounts of inorganic minerals (Kostyniuk & Likozar 2024). Depending on the approach, biorefineries aim to convert one or more of these biopolymers into targeted products. Current LCB conversion and refining can be generally classified into two categories: biochemical (fermentation and digestion) and thermochemical (combustion, pyrolysis, gasification, hydrothermal liquefaction, carbonisation, and torrefaction) (Pang 2019; Solarte-Toro et al. 2021). By and large, these conventional methods are performed using energy intensive batch processes, requiring long reaction times (hours), e.g. digestion, or large reaction systems e.g. fluidised bed pyrolysis, both of which contribute to high production and operational costs (Elhassan et al. 2025; Wiranarongkorn et al. 2023). This could prove a hindrance to large scale industrial applications given the large volumes of LCB that would need to be refined to economically produce significant volumes of important bio-derived platform chemicals (Patel et al. 2025). In contrast, a continuous processing of LCB could improve the overall techno-economics of LCB valorisation. In recent years, thermomechanical processes have emerged as an environmentally friendly alternative for biomass conversion that can efficiently breakdown LCB, avoids the use of large quantities of solvents, and offering energy efficiencies (Calcio Gaudino et al. 2022).

A promising technology in this field is twin-screw extrusion and reactive extrusion. Already well established in the polymer and food industries, extrusion is a continuous process in which solid, semi-solid or liquid materials are processed along rotating screws in a heated extrusion barrel through a confined space to achieve intense mixing. Reactive extrusion extends this concept by using the extruder as a reaction vessel to complete chemical reactions and has been demonstrated in the synthesis and modification of polymers (Hopmann et al. 2017; Lease et al. 2023; Moad 2011). The versatility of extrusion lies in the easy adjustment of key parameters such as feed rate, screw and barrel length, operating temperatures, and screw rotation speed. Furthermore, the screws are modular, allowing different sections or zones to facilitate specific thermomechanical actions. Extrusion technology is well understood and implemented at a commercial scale, leaving few barriers to entry for new operations. Consequently, reactive extrusion as a thermomechanical approach offers a rapid, continuous approach with short residence times (minutes) and easy integration into downstream processes.

Despite its success in polymer processing, reactive extrusion remains underutilised in biomass valorisation. Currently, biorefineries have largely employed extrusion systems as a pre-treatment step at temperatures between 50 °C and 225 °C to modify the biomass prior to a secondary treatment (Duque et al. 2017; Karunanithy & Muthukumarappan 2013; Konan et al. 2022). Because the high shear environment of the extruder is known to be able to separate individual wood fibres; much work has been devoted to the optimisation of key extruder parameters to achieve this goal (Hietala et al. 2011; Karunanithy & Muthukumarappan 2011). For example, the fibrillation of Douglas fir was investigated using a combined hot-compressed water extraction and twin-screw extrusion as a pre-treatment method prior to enzymatic saccharification (Gu et al. 2019; Lee et al. 2009). Higher temperature (> 200 °C) extrusion of LCB is also viable, with torrefaction of *P. radiata* bark for solid biofuels (Cooke-Willis et al. 2024), and the conversion of lignocellulosic waste to levoglucosenone using an extrusion-based catalytic pyrolysis system among recent achievements (Allais 2023). However, its application to sawdust for the production of biochemicals or chemical intermediates remains largely unexplored, representing a key knowledge gap that this study addresses. Although the extraction of hemicellulose from poplar wood via extrusion was first demonstrated in the 1990s, the use of sodium hydroxide (NaOH) solution would preclude any use in a modern biorefinery setting that would favour a solvent-free approach (N'Diaye et al. 1996).

Herein, we discuss using a solvent-free twin-screw extrusion system to produce a biochemical crude liquor from LCB obtained from *P. radiata* sawdust. We show how parameters such as screw design, screw speed, moisture content, and reaction temperature influence the yield and composition of the obtained extrusion liquor. To our knowledge, this is the first research that focuses exclusively on the chemical makeup of the liquor produced using a solvent-free wood-only extrusion process. This work demonstrates that reactive extrusion can offer a fast, solvent-free alternative for producing aqueous biochemicals, with the potential flexibility to adapt to different feedstocks and target products.

## Materials and methods

### Materials

*P. radiata* sawdust was sourced from Red Stag, Rotorua, New Zealand. The sawdust was sieved to obtain particles of less than 3 mm before extrusion to ensure good feeding and processability. Particles that did not pass through this sieve were not used for extrusions. The moisture content (MC) of sawdust was determined in triplicate using a Mettler Toledo High-Performance Halogen Moisture Analyser HX204 (Mettler Toledo, Switzerland) using the standard oven dry basis method ASTM D4442-20 and the ASTM D6980-17 method for the moisture analyser with temperature adjusted for wood (ASTM 2017, 2020).

Nitrogen for the reactive extrusion setup was supplied using a High Purity Nitrogen Generator NM30LA (Peak Scientific Instruments Ltd.).

### Reactive extrusion setup

**Fig. 1:**
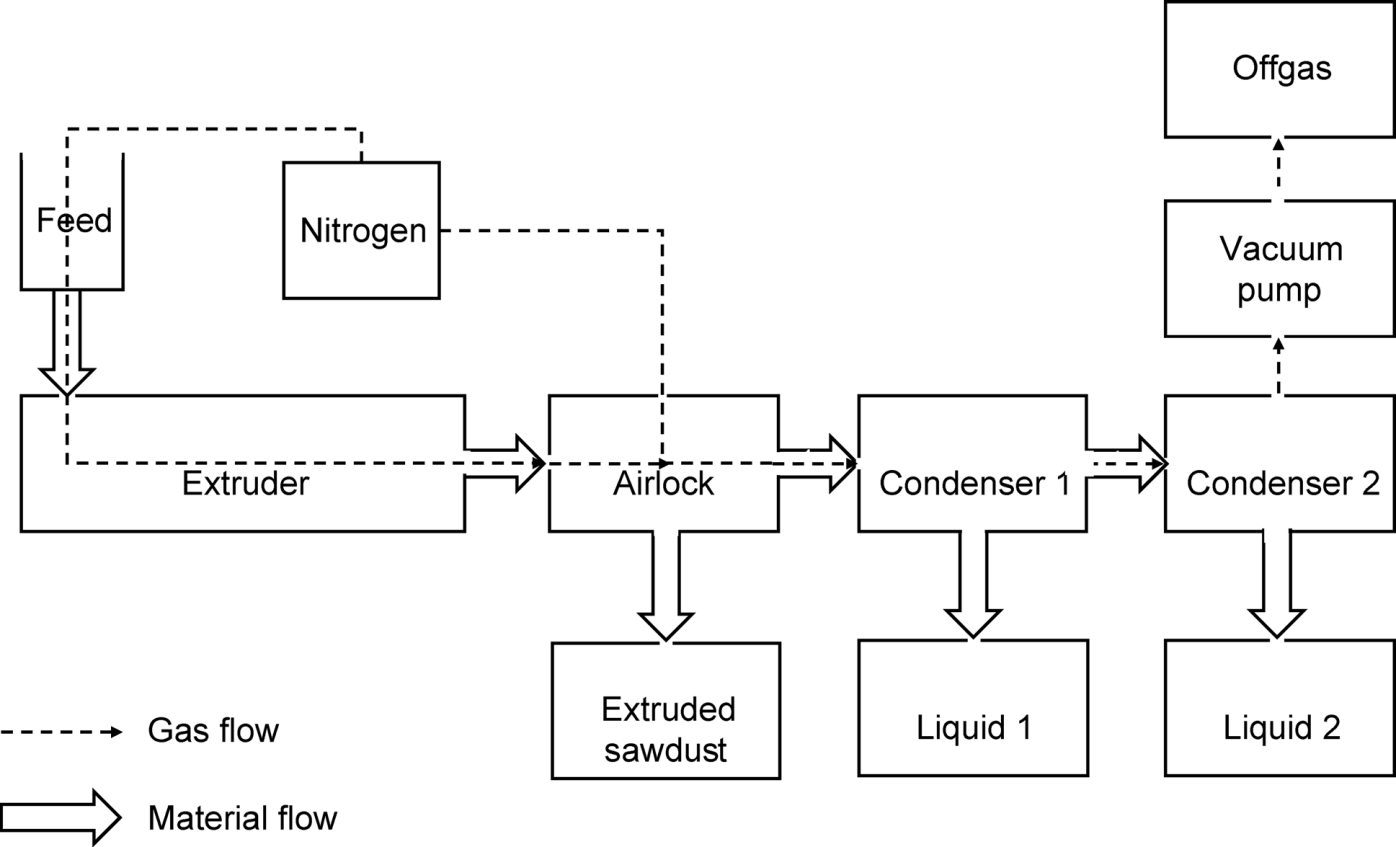
Setup of the reactive extruder

Reactive extrusion was done using an LTE26-40 26 mm co-rotating twin screw extruder by Labtech Engineering Co. LTD. (Thailand) with a 40:1 length: diameter (L/D) ratio. The sieved sawdust was fed using a K-Tron K-ML-SFS-KT20 gravimetric feeder. A 3-chamber, purpose-designed airlock setup was fitted to the die-end of the extruder to facilitate the separation of processed solids and vapours (Cooke-Willis et al. 2024). All experiments were run under an inert atmosphere to prevent the combustion of sawdust. Nitrogen was fed through two ports, the first connecting the sawdust gravimetric feeder to the extruder's main feed port and the second to the 3-chamber airlock to remove any potential air from the transit zone between solid discharges. Figure 1 depicts a schematic of the overall reactive extruder setup.

### Reactive extrusion experiments

The nitrogen, with an inflow rate of 16 L/minute, and vacuum pump were started about 20 min before each trial to purge the extruder and airlock system. The vacuum pump was kept running for the duration of the experiment to direct steam and condensable compounds into Condenser 1 and 2. The extruder was directly heated using electric heating elements controlled via thermocouples. The first section was only heated to 80 °C to avoid steam build-up in the feeding section during the extrusion trials. All other sections were heated to the desired processing temperature. Screws were set to the desired rotation prior to feeding the sample. Sawdust was fed at a rate of 1.5 kg/h for a total time of 0.5 h for all trials. The extruded sawdust was collected below the airlock chamber, while condensable compounds were collected in Condenser 1 (cooled to 60–90 °C) and Condenser 2 (cooled to 19–25 °C). Only the solid and liquid samples were collected and analysed. The extrusion liquors from Condensers 1 and 2 were combined, filtered to remove fine solid particles, and stored at -80 °C prior to chemical characterisation to avoid changes in their chemical composition, e.g. degradation of individual compounds due to low pH.

### Parameter screening runs

The effect of four parameters, viz*.* screw design, screw speed, moisture content, and temperature, on the twin-screw extrusion of sawdust was investigated in this work.

#### Screw design

Two extruder screw designs were tested to assess their feasibility for biomass processing. Both screw designs had four single-flight transport elements at the beginning to ensure good feeding of the sawdust, and a 39 mm 0.5 diameter (D; 13 mm) pitch discharge element fitted at the airlock end. All other transport elements, apart from those adjusted, were standard double-flight conveying elements with 1 D pitch. Full details of the screw design can be found in the ESI (Table S1 and S2).

**Fig. 2:**
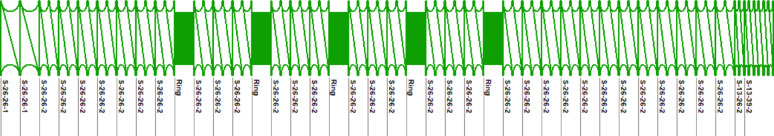
Extrusion screw configuration A (extrusion direction left to right). Green solid blocks represent spacer elements

Screw configuration A included five-purpose design 1 D (26 mm) spacers with no conveying or kneading action to increase the gap between the barrel and the screw (Fig. 2).

**Fig. 3:**
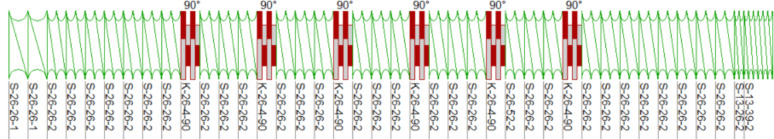
Extrusion screw configuration B (extrusion direction left to right). Red blocks represent kneading elements

Screw configuration B included six 1 D (26 mm) kneading sections, each comprising four 2-lobe kneading discs (Fig. 3).

Screw design experiments were conducted at two different screw speeds (100 rpm and 300 rpm). The constants during this test were extrusion temperature (275 °C), sawdust feed rate (1.5 kg/h), and time of extrusion (0.5 h). Sawdust processability was used as the output variable.

#### Moisture content and screw speed

Nine experiments were performed in duplicate (Table 1) to investigate the combined effect of screw design and approximate moisture content on the amount of chemicals produced in the liquor. Samples of extrusion liquors obtained from the experiments were freeze-dried and the amount of g_(biochemicals)_ per g_(liquor)_ was calculated and used as the output variable for this work.

1 $$\begin{array}{c}Biochemicals (weight \%) = \left(\frac{{w}_{biochemicals} }{{w}_{liquor}}\right) \times 100\end{array}$$

where w_liquor_ represents the weight of the liquor freeze-dried and w_biochemicals_ is the residue after freeze-drying. The validity of the freeze-drying method was confirmed via Karl-Fisher titration of selected liquors using an 870 KF Titrino Plus by Metrohm, applying the volumetric technique and the KFT Ipol measuring mode. The data was analysed by ANOVA (two-factor with replication, *p* < 0.05) to test for statistical significance. Constants for this set of experiments were screw design (B), feed rate (1.5 kg/h), and time of extrusion (0.5 h). For a feed rate of 1.5 kg/h, the residence time was determined to be 82 s at 50 rpm, 46 s at 100 rpm, and 18 s at 300 rpm.

**Table 1:** List of experiments run for determining the screw speed and moisture content of sawdust extrusion

Run #	Temperature, °C	Screw speed, rpm	Moisture content, (w/w) %
1	275	50	30 ± 3
2	275	50	40 ± 3
3	275	50	50 ± 3
4	275	100	30 ± 3
5	275	100	40 ± 3
6	275	100	50 ± 3
7	275	300	30 ± 3
8	275	300	40 ± 3
9	275	300	50 ± 3

#### Temperature

The temperature screening experiments were conducted at 275 °C, 325 °C, and 375 °C. The constants for this reaction were: screw design (B), screw speed (50 rpm), moisture content (50 ± 3%), feed rate (1.5 kg/h), and duration of extrusion (0.5 h). The amount of biochemicals produced per g of liquor after freeze drying, using Eq. 1, was used as the output variable.

### Analytical methods

#### pH of biochemical liquors

The pH of the biochemical liquors was measured immediately after extrusion and filtration using a Denver Instrument UltraBASIC pH/mV meter (UB-10).

#### Volatile fatty acid (VFA) analysis

The VFA content in the biochemical liquors was measured using an Agilent gas chromatography flame ionisation detection (GC-FID) 7890 instrument using an in-house developed method (Ranganathan et al. 2025). Methanol, ethanol, acetic acid, propionic acid, iso-butyric acid, n-butyric acid, pentanoic acid, and hexanoic acid concentrations were determined. Formic acid (3% solution) was added to correct pH, and 1-butanol (40 µL of 1000 ppm solution) was added as an internal standard. Samples were analysed using the Chromeleon™ Chromatography Data System software (ThermoFisher Scientific). All analyses were in duplicate.

#### Lignin & carbohydrate quantification of extruded sawdust samples

The Klason Lignin and Acid-soluble lignin analysis methods were based on TAPPI T222 om-02 and TAPPI Useful Method 250, respectively. Carbohydrate analysis was performed on the Klason lignin hydrolysed sample filtrate and quantified using a Dionex ICS6000 High-Pressure Ion Chromatography (HPIC) instrument (ThermoFisher Scientific) equipped with a PA10 column with eluent generation at 2 mM potassium hydroxide (KOH) (Pettersen 1991; Suckling et al. 2017). All analyses were in duplicate.

#### Thermal desorption gas chromatography-mass spectroscopy (GC–MS)

GC–MS was performed on an Agilent gas chromatograph 7890A coupled with a 7000 B triple quadrupole mass spectrometer, using a 30 m DB 624 Ultra Inert J&W Agilent Technologies column with 250 µm inner diameter and 1.4 µm film thickness. Extrusion liquors were directly analysed without further sample preparation. Samples were heated in the thermal desorption unit for 0.1 min at 64 °C, then further heated to 200 °C at a rate of 720 °C/min and held at 200 °C for 1.44 min before being injected into the cooled injection system set at −10 °C and a solvent vent injection was performed. Helium carrier gas was used at a 1.0 mL/min flow rate. The oven was set to 40 °C, ramped to 250 °C at 5 ºC/min, and then held for 20 min. The interface was set to 280 °C, and the ion source adjusted to 250 °C. The mass spectra were recorded at 0.1 scan per second with an m/z 30–400 scanning range. The chromatograms and mass spectra were evaluated using MassHunter Qualitative Analysis v 10.0, MassHunter Quantitative Analysis (Quant-My-Way) software and the NIST 2014 library (Agilent). Potential compounds were initially identified using MassHunter Qualitative Analysis v 10.0. Those with a molecular feature score higher than 80% were then evaluated using Agilent MassHunter Quantitative Analysis deconvolution algorithm couple with the NIST14 library. The identification of compounds is considered tentative. All peaks were manually integrated, and the mass percentage of each compound was determined by individual peak area against the total area of all peaks found in the sample. Samples were run in duplicate.

#### Solution state nuclear magnetic resonance (NMR)

Solution state NMR spectra were obtained with a Bruker Avance NEO 600 MHz NMR spectrometer using a dual-channel BBO iProbe tuned to the appropriate frequencies for ^1^H (600.36 MHz) and ^13^C (150.96 MHz). 2-dimensional Heteronuclear Single Quantum Coherence (2D HSQC) spectra were obtained using the "hsqcedetgpsisp2.3" pulse program with a spectral width of 9615.39 Hz and 30,195.15 Hz for proton and carbon, respectively, and a pulse delay of 1.5 s. Chemical shifts were referenced to the residual solvent peaks (H/C 7.26/77.13 for chloroform (CDCl_3_), 2.50/39.52 for dimethyl sulfoxide-d6 (*d6*-DMSO)). Attempts to analyse the whole liquor by dissolving it in a polar deuterated solvent (e.g. *d6*-acetone, *d6*-DMSO) resulted in significant overlapping, broadening of peaks, and loss in resolution of individual compounds. Cleaner spectra were obtained using the following method: Volatile chemicals in the liquor were extracted into CDCl_3_ via vortex mixing, approximately 5 mL of liquor with 1 mL of CDCl_3_ for 1–2 min. The CDCl_3_ fraction, containing phenolics and some furanic compounds, was separated and passed through a drying column of sodium sulphate prior to analysis. The remaining water-soluble portion of the liquor, containing oligomeric sugars, lignin, and highly water-soluble furanic compounds that were not extracted into CDCl_3_, was freeze-dried to concentrate the remaining biochemical residue and then dissolved in *d6*-DMSO for analysis.

#### Solid state NMR

Cross-Polarisation Magic Angle Spinning (CP MAS) ^13^C NMR spectra were obtained using a Bruker Avance NEO 500 MHz NMR spectrometer using a dual-channel 4 mm H/X MAS iProbe tuned to 125.76 MHz. MAS rotation rate was set to 10 kHz, relaxation delay (D1) of 3 s, and acquisition time of 0.0276 s, with a total of 1024 scans.

## Results and discussion

### Parameter screening – screw design

The highly confined environment of the twin-screw extruder provides "micro areas", where the combination of high shear, pressure, and temperature is instrumental in breaking down the structure of LCB. To control the energy exerted on the biomass, screw design was the first parameter varied in this study. Our initial hypothesis was that the conveying elements alone would be sufficient to provide the shear required to break down the small sawdust particles. With this in mind, two designs were evaluated: A (with spacer elements, Fig. 2) and B (with kneading elements, Fig. 3). Screw design A introduced spacer elements to provide relaxation for the material and allow pockets of superheated steam to enhance hydrothermal interaction with the sawdust. Screw design B added kneading elements to increase the intensity of the shear effect on the sawdust (Gu et al. 2019; Karimipour-Fard et al. 2024). Initial experiments were conducted with a screw speed of 100 rpm. Screw design B showed good processability. However, screw design A performed very poorly, with frequent blocking of the extruder and inconsistent operation. A second set of experiments was conducted using a screw speed of 300 rpm to push the material through quicker. However, this did not improve the blockage problem. In contrast, screw design B again performed well at this speed. The poor performance of screw design A was due to sawdust build-up in the spacer elements despite feeding particles that were less than 3 mm in size. The success of screw design B can be attributed to the significant shear force that could only be applied via kneading elements, combined with agitation that, unlike the spacer areas in design A, avoided stagnation, overpacking and fusion of wood particles. Hence, screw design B was chosen for further tests.

### Moisture content and screw speed

**Fig. 4:**
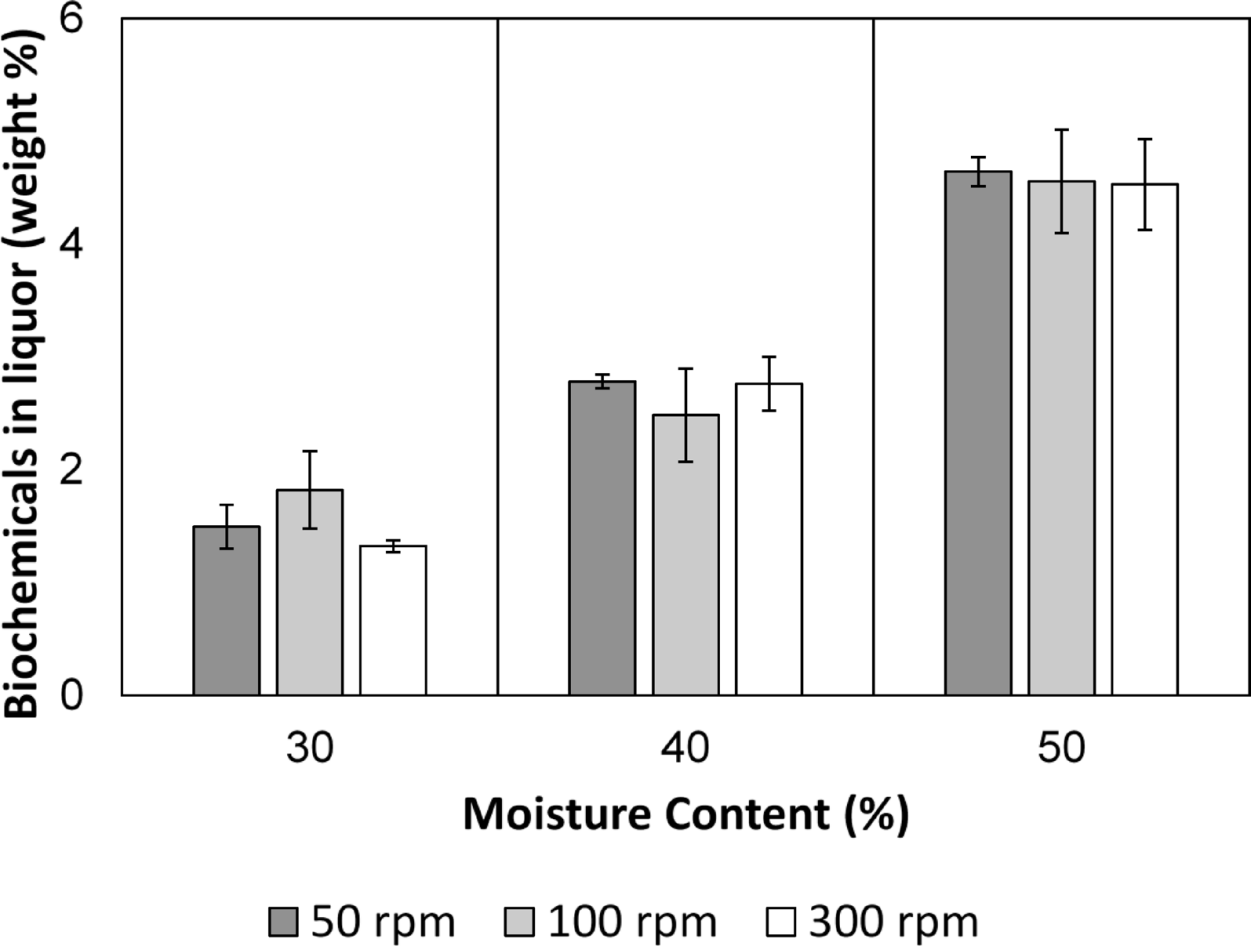
Freeze-dried biochemical content in liquor after thermomechanical degradation of sawdust as a function of moisture content and screw speed

Following the choice of screw design B, the next set of parameters screened were moisture content and screw speed. Moisture content can affect the extruding process by changing the friction between the material, the barrel and the screws, affecting the generation of shear heat and the thermal softening of biomass, and the composition of obtained products (Guiao et al. 2022; Zhao et al. 2019). Furthermore, hydrothermal force can be generated when moisture in the biomass is flashed off at the end of the extruder die (Karunanithy & Muthukumarappan 2013). Freshly obtained sawdust was visibly wet with a moisture content of typically 55%. Ideally, biomass with this maximum moisture content could be used to maximise the effect of superheated water within the extruder barrel. However, feeding this sawdust proved difficult with blockages in the feed pipe and extruder throat. Briefly air-drying the sawdust to a moisture content of 50%, presumably through evaporation of surface-bound water, improved the processability and was set as the highest moisture content tested. Two other moisture contents (30% and 40%, obtained by further air-drying of the sawdust) were also tested. The screw speed was also varied across three levels—50 rpm, 100 rpm and 300 rpm – matching the speeds used in the screw design experiments, with 50 rpm included as an additional lower-speed condition. The weight percentage of biochemical content in the liquor (determined gravimetrically by freeze-drying, Eq. 1) was used as the output variable for these runs. Temperature (275 °C) and operating time (0.5 h) were maintained constant, and the results of these experiments are shown in Fig. 4.

Two things are evident from the figure. The amount of biochemicals in the liquor post-extrusion was directly proportional to the moisture content in the biomass, and screw speed/residence time did not influence the amount of biochemicals in the liquor. Statistical analysis (*p* < 0.05) of the results confirms that the moisture content had a significant effect on the biochemical content (*p* = 0.004). Hence, sawdust with a high moisture content of 50% was carried forward to temperature screening. Surprisingly, the screw speed had no significant effect on the biochemical content (*p* = 0.893; refer to ESI for two-way ANOVA). This presented a dilemma as to the ideal screw speed. In the end, a screw speed of 50 rpm was chosen based on several factors. Firstly, more particle size reduction was visually evident in sawdust extruded at 50 rpm compared to 100 rpm and 300 rpm, indicating better shearing. Secondly, low screw speed favours an increase in specific mechanical energy, which improves the breakdown of polymeric biomass components into chemicals (Gu et al. 2019). Thirdly, high screw speeds increase friction torque which decreases specific mechanical energy (Godavarti & Karwe 1997). Since the focus of the work was to maximise the amount of chemicals, a lower specific mechanical energy runs counter to our overall goal.

### Temperature

**Fig. 5:**
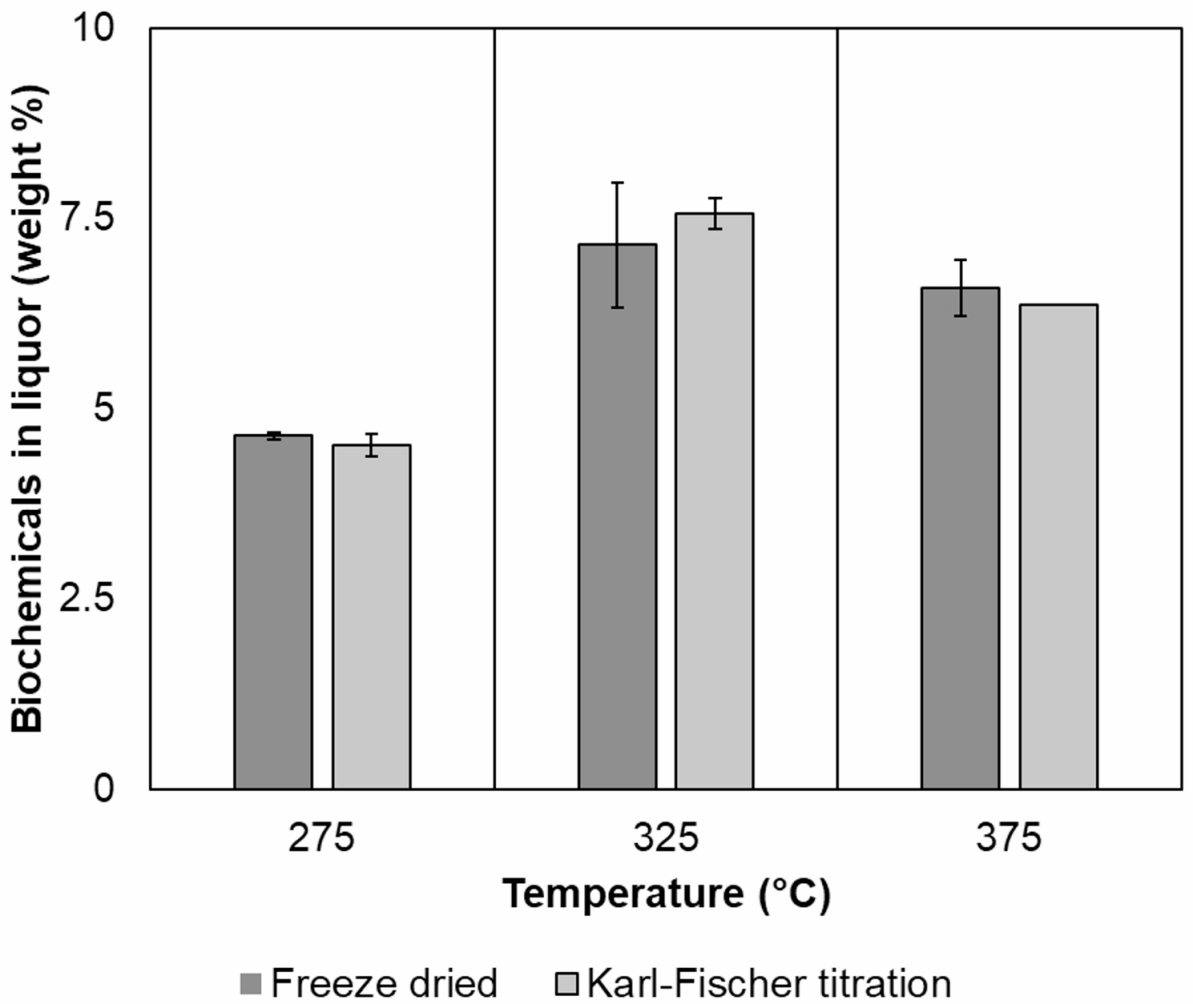
Biochemical content in the liquor determined by Karl-Fisher titration (light grey) and freeze-drying depending on the extrusion processing temperature. The error bars represent the standard deviations of the observations (n = 3 replicates)

The final screening parameter was the extruder barrel temperature. The results obtained for extruding sawdust with 50% moisture content, screw design B, and screw speed of 50 rpm, with varying temperatures, are shown in Fig. 5.

Freeze drying is the recommended drying method for temperature-labile substances. However, the loss of volatiles from any solution while drying is inevitable. Hence, Karl-Fisher titration analysis was performed on the extrusion liquors to determine the total biochemical content and prevent any erroneous estimation of biochemicals. As evident from the figure, the difference in the biochemical content in the extrusion liquor by freeze-drying and Karl-Fischer titration analyses is well within the range of error. Hence, it can be concluded that freeze-drying did not remove any volatiles from the liquor and can be used to estimate biochemical content in the liquid. Approximately 4.5% of chemicals were obtained for the 275 °C run, while the 375 °C run yielded 6.5% of chemicals (on a dry basis). The highest yields were generally obtained at 325 °C, which yielded 7.2–7.5% of chemicals. The drop in chemical content in the liquor at 375 °C might be attributed to the adsorption of chemicals onto the solid residue (Cooke-Willis et al. 2024; Song et al. 2021).

### Proposed mechanism of extraction using reactive extrusion

The overall biochemical yield in the liquor appears to be independent of screw speed and residence time but is highly influenced by the moisture content of the sawdust. This strongly indicates that the mechanism by which reactive extrusion is extracting chemicals is via a rapid volatile release. The reactive extruder achieves this via the combination of mechanical and thermal processes. The high shear environment has several effects on the sawdust. Firstly, there is a reduction of particle size of the sawdust, leading to an increase of surface area allowing for better extraction. Secondly, at the molecular level there is disruption of the sawdust fibrils and plant cell structure. When combined with rapid heating, the water trapped within the sawdust volatilises, breaking open the already weakened fibrils and cell wall, allowing the release of biochemicals. Released water can also participate in further depolymerisation of hemicellulose or the formation of acetic acid via hydrolysis of hemicellulose acetate groups which further assists the breakdown of biomass into chemicals such as furfural (Shankar Tumuluru et al. 2011). A higher initial moisture content would favour the depolymerisation process which explains why the initial moisture content of the sawdust has a significant effect on biochemical yield.

## Analysis of extrusion solids fraction

### Solid state NMR

**Fig. 6:**
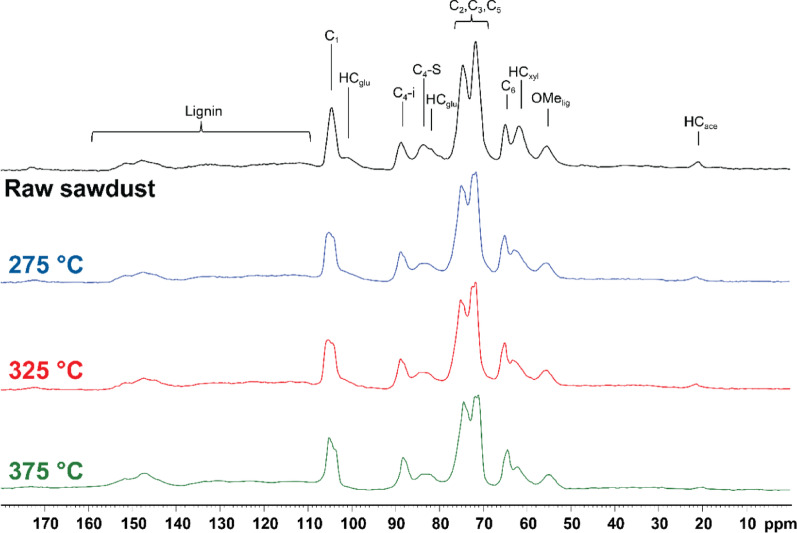
CP MAS ^13^C solid state NMR spectra of raw *P. radiata* sawdust and sawdust extruded at 275 °C, 325 °C, and 375 °C

CP MAS ^13^C solid-state NMR spectra of raw *P. radiata* sawdust and the sawdust extruded at 275 °C, 325 °C, and 375 °C are shown in Fig. 6. The spectrum of raw *P. radiata* can be roughly divided into two domains, namely a lignin domain from 110 to 160 ppm and a predominantly carbohydrate domain from 20 to 110 ppm consisting of cellulose and hemicellulose, along with one resonance from the lignin methoxy at 55 ppm. The assignment of lignin, cellulose and hemicellulose signals in *P. radiata* has been reported previously (Hill et al. 2013). Initial inspection of the spectra of the extruded sawdust reveals that much of the carbohydrate domain remains at the end of the extrusion process. Even up to an extrusion temperature of 375 °C, significant peaks from cellulose in the 70–105 ppm ranges are still clearly visible. There appears to be little change in the lignin region. This region is also where any resonances from potential biochar formed from the repolymerisation of sugar monomers and their dehydration products, like furfurals, would likely be observed (Baccile et al. 2014; Titirici et al. 2008). However, significant degradation of the carbohydrates into biochar, even up to extrusion temperatures of 375 °C, is not readily visible. Despite these temperatures being typical of torrefaction, the lack of significant biochar signals reflects the milder conditions of extrusion because of one, the high moisture content of our feedstock and, two, the short residence time of the extruder compared to typical torrefaction conditions. This is an encouraging result because our goal was to extract chemicals and not make biochar. Despite this, there are clear signs of the effect of the extrusion process visible in the carbohydrate region. The most obvious of these is the increased splitting of the large, broad peaks between 70 ppm and 80 ppm, attributed to the C_2_, C_3_ and C_5_ carbons in cellulose, and the peak at 102–108 ppm from the C_1_ carbon in cellulose. This is likely due to changes in the crystallinity of the cellulose caused by the mechanical interaction of the biomass with the twin-screw (Barakat et al. 2014). When comparing the sawdust extruded at different temperatures, there is a clear decrease in intensity of signals at 21.1 ppm, 61.7 ppm, 81.9 ppm and 101 ppm, with a slight decrease at 275 °C compared to raw biomass, to almost absent and/or completely overlapped by cellulose signals at 325 °C and 375 °C. The signals at 21.1 ppm, 61.7 ppm, 81.9 ppm, and 101 ppm have been previously assigned as coming from hemicellulose acetyl, xylans, and glucomannans (Liitiä et al. 2003; Liu et al. 2015), and their disappearance is a clear indicator of the breakdown of hemicellulose under the reactive extruder conditions. Another notable feature is the decrease in the signal at 83.9 ppm, which has previously been attributed to cellulose on the surface of the biomass fibril (Foston 2014; Hill et al. 2013; Melkior et al. 2012). This could be expected since the surface cellulose in a fibril would be the most vulnerable to the heat and mechanical pressure of the twin-screw. Comparatively, the signal at 89.3 ppm, which is typically assigned as coming from cellulose from within the interior of the fibril, remains largely unchanged.

### Lignin and carbohydrates analysis

**Fig. 7:**
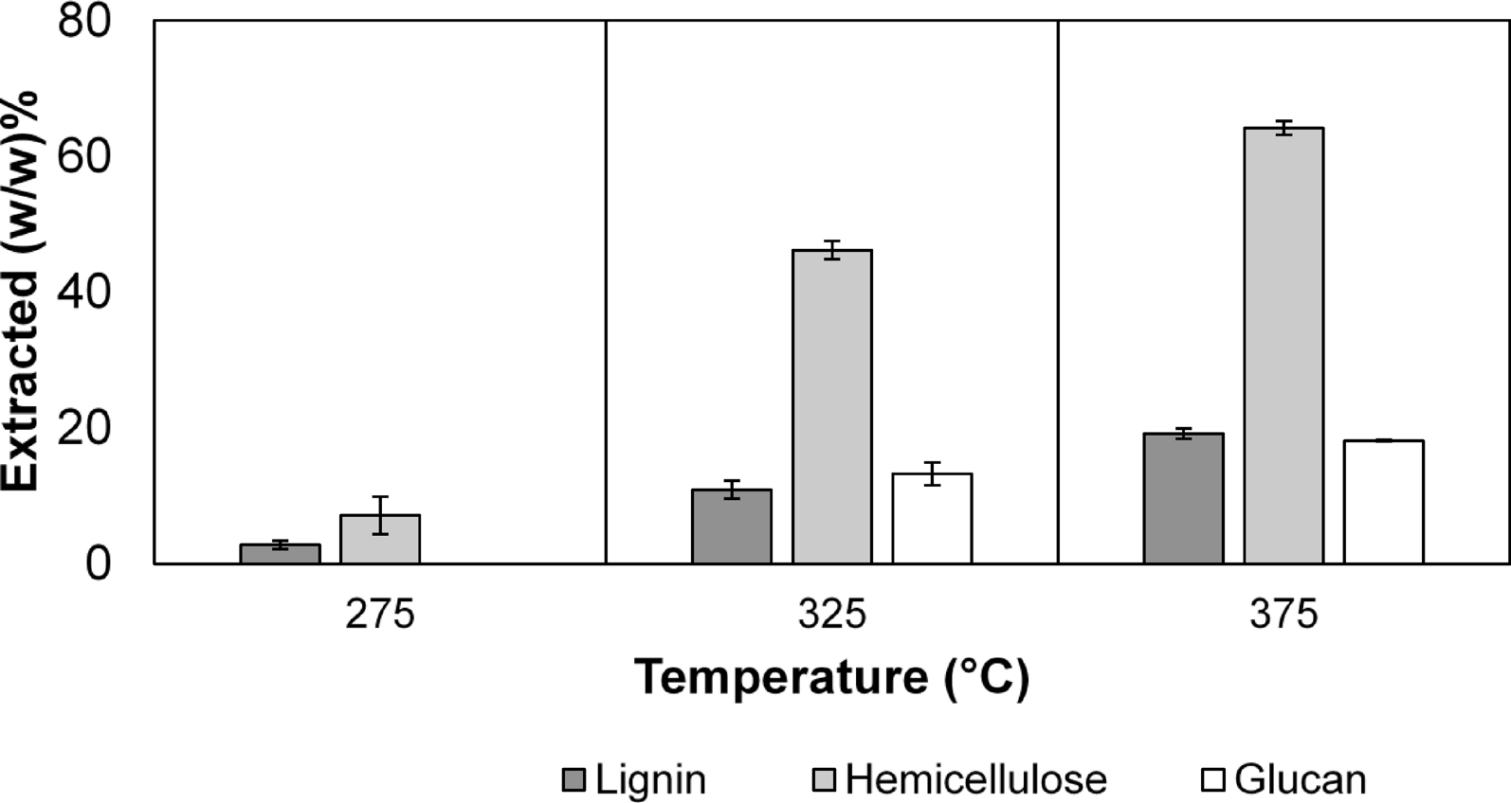
Total lignin, hemicellulose and glucan extraction percentage for the three different temperatures (275 °C, 325 °C, and 375 °C) at constant screw speed (50 rpm), and moisture content (50%)

*P. radiata* sawdust used in this work, analysed by Tappi standards (TAPPI 1991, 2002), had the following biochemical breakdown: 30.38% Klason lignin (KL), 0.64% Acid soluble lignin (ASL), 1.32% arabinans (Ara), 5.11% xylans (Xyl), 2.92% galactans (Gal), 9.75% mannans (Man), and 41.76% glucans (Glc), similar to reports in literature (Miguel et al. 2015; Santos et al. 2024). The results obtained post-extrusion at three different temperatures are shown in Table 2 as individual mass percentages of the components of lignin, cellulose, and hemicellulose extracted. Figure 7 denotes total lignin (as a sum of KL and ASL), hemicellulose (as a sum of arabinans, xylans, galactans, and mannans), and glucan (either from cellulose or hemicellulose) extraction. As seen in Fig. 7, at 275 °C, a total of 2.81% of lignin was extracted along with 7.13% of hemicellulose. It is worth mentioning that no glucan extracted from the biomass at this temperature. However, at 325 °C, 10.89%, 46.14%, and 13.27% of lignin, hemicellulose and glucan was extracted, respectively. This was significantly lower than the amount of lignin (19.15%), hemicellulose (64.15%), and glucan (18.14%) extracted from the reactive extrusion process at 375 °C. Table 2 shows that an increase in the temperature of the extrusion process increases the extraction of lignocellulosic components. Arabinans were extracted the most at all tested temperatures, followed by xylans, galactans, and mannans, which represent the hemicellulose fraction that matches the literature for hemicellulose extractions from softwoods (Sermyagina et al. 2022). Cellulose and lignin are recalcitrant, owing to their role in nature, i.e. preserving the chemical and structural integrity of the biomass. Cellulose is an unbranched and mixed polymer of crystalline and amorphous nature embedded deep into the lignin and hemicellulose layer, comprising glucose monomers. The combination of its location and its chemical inertness in water (at temperatures below 300 °C) can be the reason glucans have not been extracted at 275 °C (Nair et al. 2023; Petridis & Smith 2018). An increasing trend in glucan extraction was observed when the temperature was raised. Lignin is a polyphenolic polymer that stiffens the cell wall, and even though Klason lignin degradation started at 275 °C, the acid-soluble lignin extraction was not possible at 275 °C. However, Klason-lignin and acid-soluble lignin extraction increased at 325 °C and 375 °C.

**Table 2:** Extraction of different lignocellulosic fractions expressed as mass fractions for various operating conditions

Operating conditions (T °C, rpm, MC %)	KL extracted (w/w) %	ASL extracted (w/w) %	Glc extracted (w/w) %	Ara extracted (w/w) %	Xyl extracted (w/w) %	Gal extracted (w/w) %	Man extracted (w/w) %
275, 50, 50	2.90 ± 0.63	0	0	18.47 ± 2.35	8.47 ± 3.43	6.24 ± 2.39	5.15 ± 2.59
325, 50, 50	10.93 ± 1.49	9.10 ± 5.70	13.27 ± 1.69	67.26 ± 0.61	52.29 ± 1.10	52.15 ± 1.51	38.25 ± 1.54
375, 50, 50	19.14 ± 0.71	19.50 ± 2.67	18.14 ± 0.04	79.15 ± 1.15	67.32 ± 0.67	69.86 ± 0.83	58.73 ± 1.22

MC – moisture content, KL – Klason lignin, ASL –acid-soluble lignin, Glc – glucan, Ara – arabinan, Xyl – xylan, Gal – galactan, Man—mannan

## Analysis of extrusion liquid fraction

### Volatile fatty acid (VFA) analysis of liquor

The liquor obtained from sawdust extrusion is usually a dark brown solution with a distinct odour of burnt caramel. All liquors were acidic, with the liquor extracted at 275 °C having an average pH of 2.7, and the liquors extracted at 325 °C and 375 °C having an average pH of 2.3. The low pH of the liquors is due to the presence of large quantities of acetic acid because of the breakdown of hemicellulose. This was confirmed using VFA analysis, which also showed significant amounts of methanol, another hemicellulose by-product (Table 3).

**Table 3:** Alcohols and VFA concentration (mg/L) of selected breakdown by-products present in the extrusion liquor

Temperature °C	Methanol (mg/L)	Ethanol (mg/L)	Acetic Acid (mg/L)	Propionic Acid (mg/L)
275	753	46	645	18
325	2459	30	5675	128
375	2277	5	7913	195

### NMR analysis of liquor

#### Water soluble fraction

**Fig. 8:**
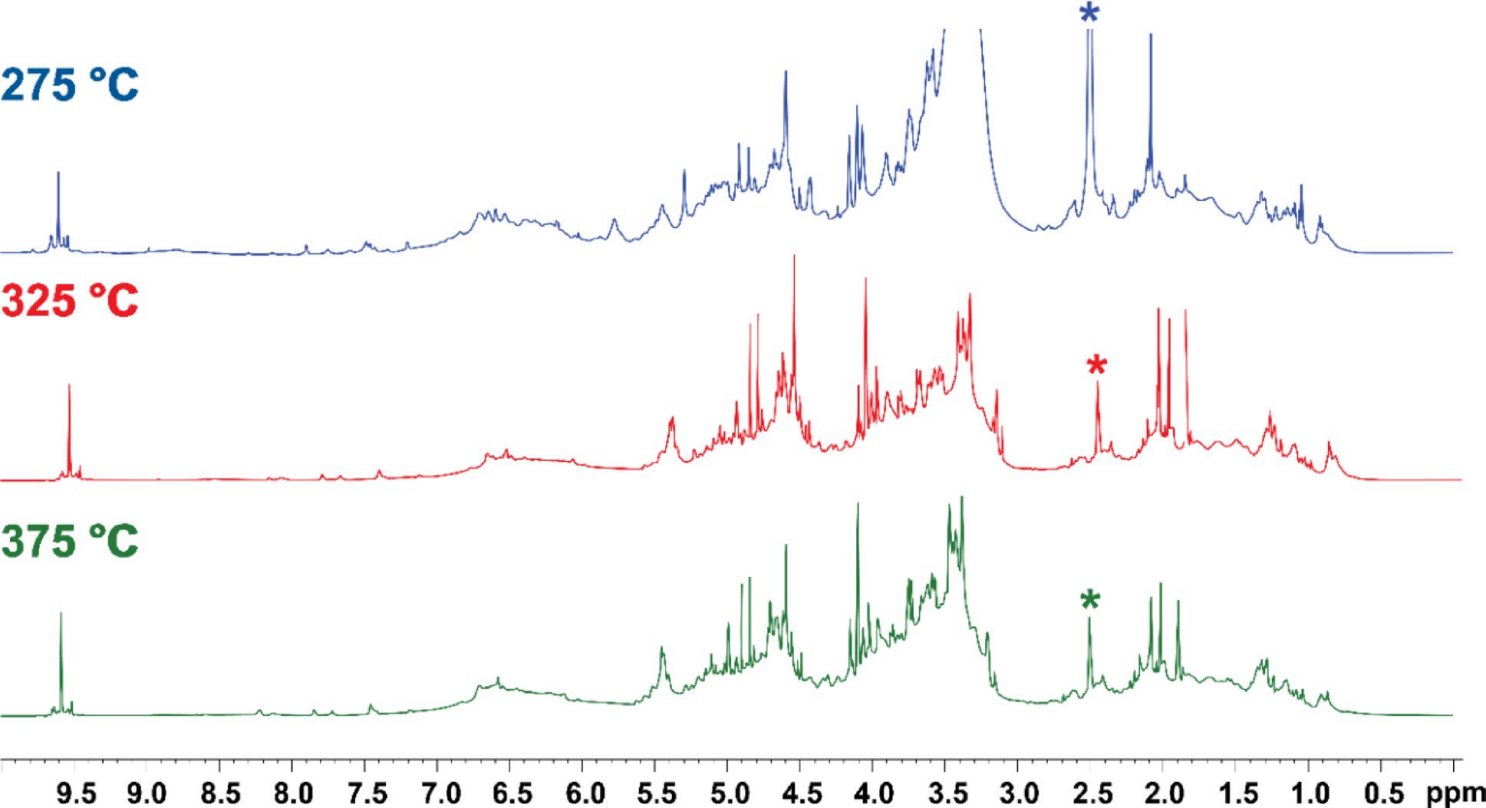
^1^H NMR spectra of the water-soluble fraction of the liquor obtained at 275 °C, 325 °C, and 375 °C. Signal from *d6-*DMSO appears at 2.50 ppm

Figure 8 shows the ^1^H NMR spectra of the water-soluble fraction of the liquor obtained at 275 °C, 325 °C, and 375 °C. ^13^C NMR spectra can be found in the ESI. The spectra are dominated by a significant region of overlapping broad peaks between 3 and 5.5 ppm, which is characteristic of sugar oligomers. Lignin breakdown products are also present as an undefined broad signal from 6 to 7 ppm. Signals possibly from furanic compounds that were not extracted into CDCl_3_ are visible between 7 and 10 ppm although no assignment of individual compounds is possible. The spectra of the 325 °C and 375 °C liquors have a series of singlets between 1.8 and 2.2 ppm, indicating that some of the sugars remain acetylated.

**Fig. 9:**
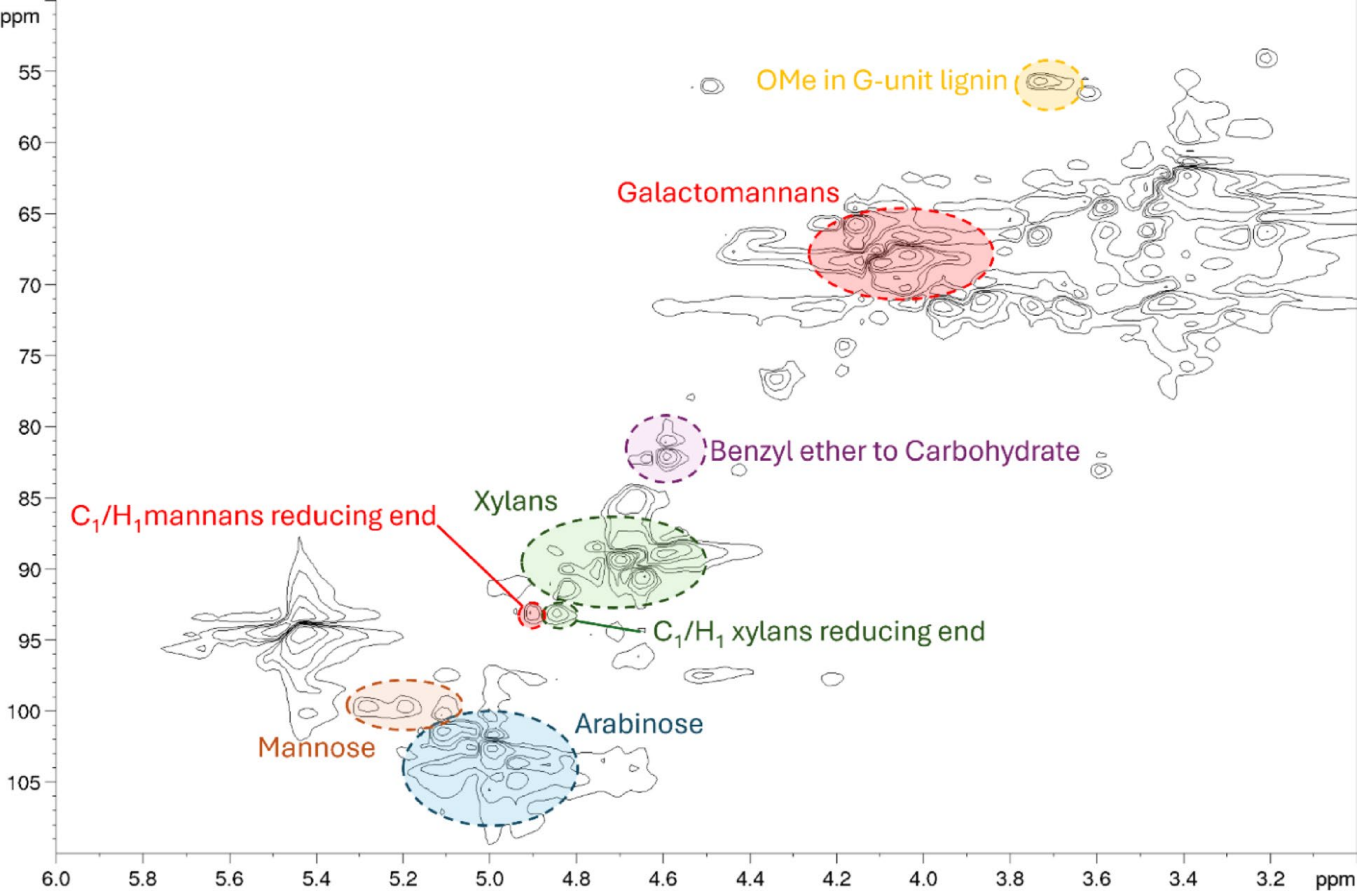
2D HSQC NMR spectrum of the water-soluble fraction of the liquor obtained with an extrusion temperature of 325 °C

2D HSQC spectra were obtained to understand the structure of the carbohydrates in the liquors. All liquors had similar HSQC spectra, in line with the similarities observed in the ^1^H NMR spectra. The HSQC spectrum of the water-soluble phase of the liquor obtained at 325 °C is shown in Fig. 9 (see ESI for spectra from 275 to 375 °C liquors), and assignments of correlations were made with literature precedent where possible. The majority of carbohydrate signals arise from hemicellulose, whereas no resolvable correlations that were potentially from glucans were visible. This is consistent with the survival of cellulose as observed in the solid-state NMR spectra and the carbohydrate analysis which showed significant extraction of hemicellulose from the extruded sawdust. Three clusters of correlations could be identified as belonging to hemicellulose carbohydrates. The first at C/H 66–70/3.9–4.2 was assigned to galactomannans (Muschin & Yoshida 2012), the second at C/H 88–94/4.6–4.9 was assigned to xylans (Teleman et al. 2000), and the third at C/H 100–108/4.8–5.2 assigned to arabinose (Kim & Ralph 2010). Small amounts of mannose correlations were visible between C/H 100–102/5.0–5.4 (Kim & Ralph 2010). The two strong signals at 4.84 ppm and 4.89 ppm in the ^1^H NMR spectra could be assigned as the C1/H1 in α-D-xylopyranoside of xylans reducing end, and C1/H1 in α-D-mannopyranoside of mannans reducing end, respectively based on the 2D HSQC data (Giummarella & Lawoko 2017). Signals arising from lignin also overlap with carbohydrates in this region. The two that could be tentatively identified were the methoxy group attached to the G-unit of lignin at C/H 55/3.71 and the benzyl ether/carbohydrate linkage at C/H 82/4.59 (Giummarella & Lawoko 2017). This benzyl ether/carbohydrate link is one of the strongest signals in the ^1^H NMR spectrum and is potential evidence of large amounts of lignin-carbohydrate complexes in the liquor.

#### Chloroform soluble fraction

**Fig. 10:**
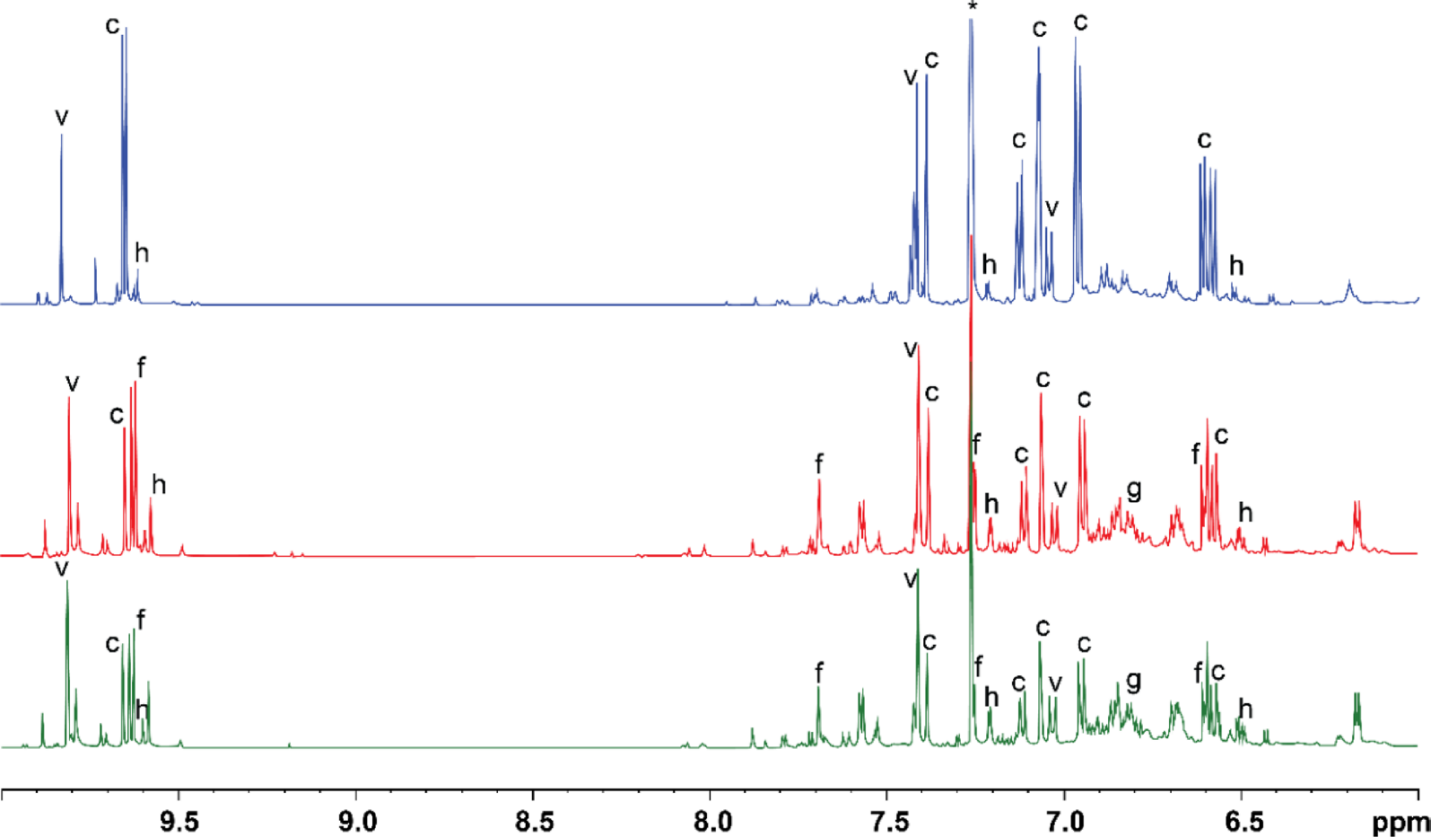
^1^H NMR spectra of the CDCl_3_ extracted chemicals from liquor produced at 275 °C(blue), 325 °C(red), and 375 °C(green). Identified compounds include coniferyl aldehyde (c), vanillin (v), furfural (f), 5-HMF (h) and guaiacols (g). CDCl_3_ residual solvent signal is at 7.26 ppm

Figure 10 depicts the NMR spectra of the CDCl_3_ fraction obtained from extrusion at 275 °C, 325 °C and 375 °C in the region between 6and 10 ppm with the assignment of some compounds based on literature precedence (Hiltunen et al. 2006; Mascal & Nikitin 2009). Full NMR spectra of the CDCl_3_ extracted liquors can be found in the ESI along with chemical shifts for assigned compounds (Table S5). The extraction of volatile chemicals into CDCl_3_ allowed the identification of major individual compounds, although complete extraction of all volatile compounds was not fully realised, as evidenced by the presence of furan signals in the water-soluble fraction NMR samples. Approximately 10% of biochemicals were extracted into CDCl_3_. The ^1^H NMR spectrum of liquor obtained at 275 °C is dominated by signals arising from vanillin and coniferyl aldehyde. Integration of the C2 protons gave a ratio of 2.6:1 coniferyl aldehyde to vanillin. Both compounds are known lignin degradation products (Sun et al. 2018). In particular, coniferyl aldehyde is derived from coniferyl alcohol, the dominant C6C3 monolignol, i.e. a building block of lignin and a common product of lignin thermal decompositions, found in G-unit lignin, the major class of lignin in *P. radiata* (Jiang et al. 2023). Although lignin is the LCB biopolymer most resistant to depolymerisation, at temperatures below 300 °C, cleavage of both the α-O-4 and β-O-4 bonds linking the monomer units is possible, releasing volatile monomeric phenolics containing side chains of two or three carbons in length (Collard & Blin 2014). Only traces of potential furans or other compounds emerging from the baseline are visible. Furans, most notably furfural are derived from pentoses obtained during the rapid depolymerisation of hemicellulose. Despite 275 °C being hot enough to cause depolymerisation of hemicellulose, it is likely that the short residence time of the extruder has not allowed this reaction to go to completion, resulting in the large amounts of oligomeric sugars in the water-soluble fraction (Borrega et al. 2011). In contrast, the ^1^H NMR spectrum of the liquor produced at 325 °C and 375 °C is considerably more complex owing to the appearance of compounds resulting from the depolymerisation of hemicellulose. Furfural and 5-hydroxymethylfurfural are the two major furan compounds that appear and are easily assigned based on literature precedent. Several other unidentified furanic compounds are also visible in the 7–8.5 ppm region. Clusters of peaks also appear between 6.6 ppm and 6.9 ppm, consistent with several compounds with guaiacol backbones.

### GC–MS analysis of liquor

Although the NMR data provides a good global overview of the biochemicals in the extrusion liquors, information about the presence and relative amounts of individual compounds is lost. Hence, GC–MS analysis was conducted to evaluate the volatile monomeric compounds in the liquor. The liquor was directly injected for analysis without any other sample preparation. A selection of the major volatile chemical compounds identified in the liquors obtained at 275 °C, 325 °C, and 375 °C, along with their respective abundances, is summarised in Table 4. Raw spectra are presented in the ESI. Based on the identified likely pathway of the creation of the identified biochemicals, the reaction products were classified into lignin-origin, carbohydrate-origin, products arising from unknown side reactions, and finally, wood components.

**Table 4:** Major volatile compounds in the liquors produced at 275 °C, 325 °C, and 375 °C with relative percentages

Compound	R.T	275 °C	325 °C	375 °C	Likely origin
Furfural	16.546	3.88	6.99	21.47	Carbohydrates
2-Furanmethanol	17.440	3.42	5.49	5.74	Carbohydrates
1,2-Cyclopentanedione	20.339	0.57	3.71	3.77	Carbohydrates
3-Furanmethanol	20.684	1.04	2.79	0.89	Carbohydrates
2(5H)-Furanone	21.441	1.34	4.31	3.72	Carbohydrates
2-Furancarboxaldehyde, 5-methyl-	21.564	0.31	n.d	2.52	Carbohydrates
Oxazolidine, 2,2-diethyl-3-methyl-	23.179	1.41	6.83	7.39	Side reactions
1,2-Cyclopentanedione, 3-methyl-	24.094	1.19	3.64	2.95	Carbohydrates
Guaiacol	26.058	0.44	1.83	10.82	Lignin
Maltol	27.318	1.01	2.10	0.85	Carbohydrates
3-Heptanone, 5-methylene	28.212	1.77	5.40	2.09	Side reactions
L-.alpha.-Terpineol	29.061	n.d	n.d	0.54	Wood Component
2,3-Dihydroxybenzaldehyde	29.262	n.d	0.42	n.d	Lignin
Creosol	29.521	0.35	n.d	5.74	Lignin
Phenol, 4-ethyl-2-methoxy-	32.222	n.d	n.d	1.85	Lignin
5-Hydroxymethylfurfural	32.764	4.77	6.79	3.25	Carbohydrates
5-Acetoxymethyl-2-furaldehyde	33.522	0.18	1.09	n.d	Carbohydrates
4-Hydroxy-3-methylacetophenone	33.603	n.d	0.54	n.d	Lignin
Eugenol	34.594	n.d	n.d	2.93	Lignin
Vanillin	37.271	21.62	13.32	4.63	Lignin
Phenol, 2-methoxy-4-propyl-	38.775	5.48	4.75	2.68	Lignin
Ethanone, 1-(3-hydroxy-4-methoxyphenyl)-	39.535	2.36	3.52	1.78	Lignin
2-Propanone, 1-(4-hydroxy-3-methoxyphenyl)-	40.707	5.15	8.20	3.37	Lignin
4-(1-Hydroxyallyl)-2-methoxyphenol	41.867	0.70	n.d	n.d	Lignin
1-(4-Hydroxy-3-methoxyphenyl)butan-1-one	42.097	2.82	1.87	n.d	Lignin
Benzenepropanol, 4-hydroxy-3-methoxy-	43.806	3.74	4.51	2.53	Lignin
Coniferyl aldehyde	47.359	34.92	7.80	1.20	Lignin

The liquor obtained from extrusion at 275 °C was dominated by lignin decomposition products, which constituted about 77–79% of the total volatile chemicals. Only 16% −18% of the compounds could be attributed to the decomposition of carbohydrates, indicating that the extracted carbohydrates are likely non-volatile oligomers, as suggested by the NMR data. The phenolic compounds obtained from lignin all contain the guaiacol backbone. Coniferyl aldehyde and vanillin are the most abundant degradation products following extrusion at 275 °C, accounting for more than 50% of the organic compounds identified using GC–MS and 73% of the total lignin breakdown products, consistent with the ^1^H NMR analysis. With regards to carbohydrate breakdown, the predominant compounds are furans, such as furfural and 5-hydroxymethylfurfural, which are known products of saccharide dehydration (Collard & Blin 2014).

**Fig. 11:**
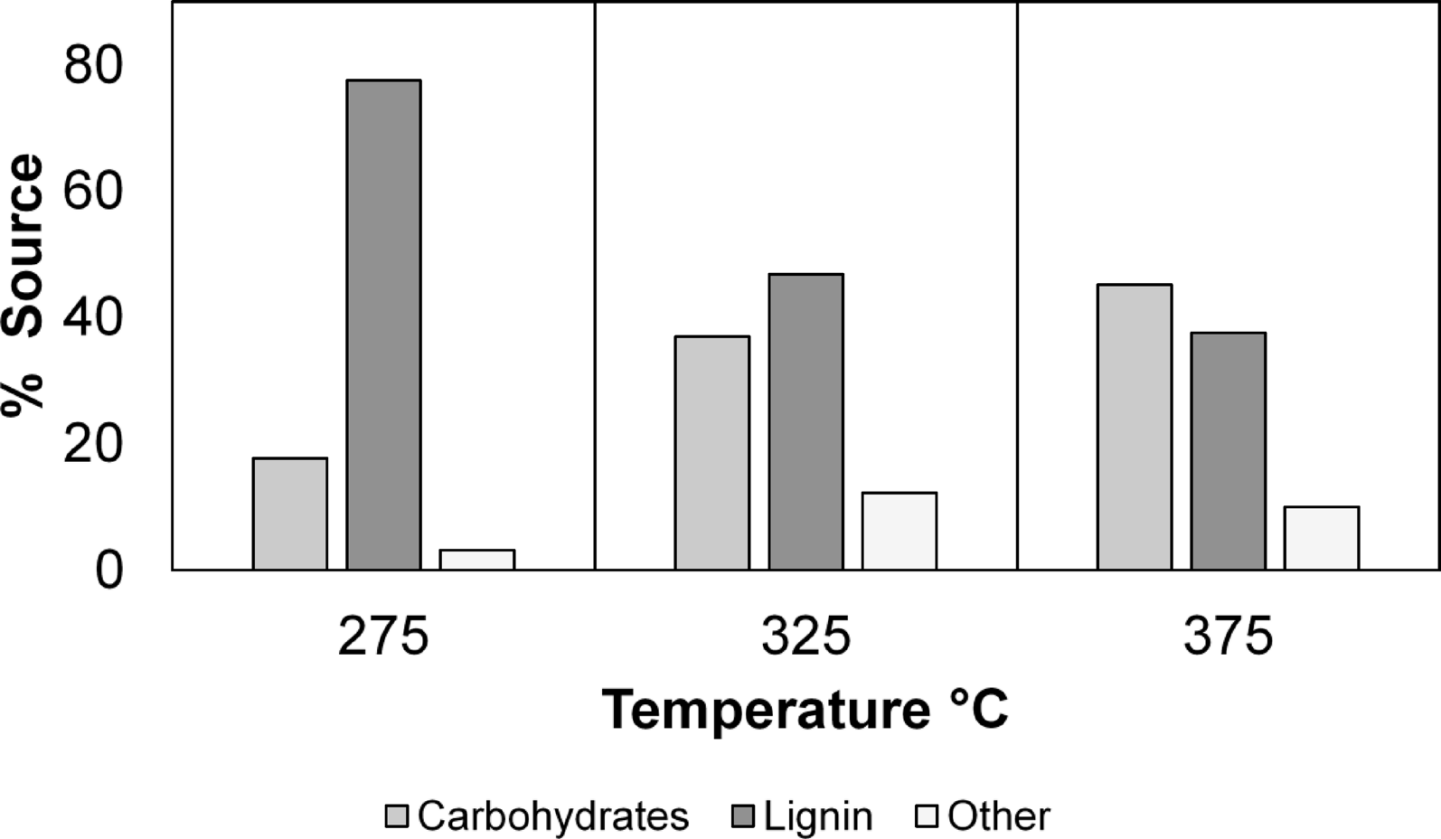
Percentage of compounds whose likely origin was from carbohydrates, lignin or other wood components and side reactions

Analysis of the extruded sawdust revealed that significant hemicellulose breakdown occurred at extrusion temperatures of 325 °C and higher. This is reflected in the composition of the volatile chemicals in the liquor, as depicted in Fig. 11. The percentage of compounds arising from carbohydrates increases from 18% of the total liquor at 275 °C to 45% at 375 °C. This increase is primarily driven by furfural, which makes up 21% of the volatile compounds in the liquor at 375 °C. Furfural is generated from the dehydration of C5 pentoses, of which hemicellulose is a major source in the form of xylose, arabinose, galactose and mannose (Namhaed et al. 2024). This reaction is catalysed by acetic acid which is a major compound in the liquor as detected in the volatile fatty acid analysis. There is also a corresponding increase in compounds derived from furfural, such as furanmethanol, furanone, and 1,2-cyclopentanedione. The higher extrusion temperature also affects the composition of the lignin compounds in the liquor. The percentage of coniferyl aldehyde drops to about 1% of the liquor obtained at 375 °C. The increase in compounds such as guaiacol, creosol, and eugenol indicates that the C_4_ chain of coniferyl aldehyde is being cleaved at higher extrusion temperatures. A similar phenomenon can explain the fall in the relative percentage of vanillin. Although significantly more hemicellulose is removed from the sawdust than lignin at higher temperatures (64% hemicellulose removed versus 19% lignin removed at 375 °C), this is not reflected in the composition of the volatile chemicals in the liquor (45% compounds from hemicellulose versus 37% from lignin at 375 °C). This is due to the extracted hemicellulose remaining as oligomers as confirmed by NMR analysis rather than decomposition to monomers and furans since these large, bulky molecules would not expect to fly in the GC–MS.

## Further discussion

The overall aim of this study was to obtain an aqueous phase rich in biochemicals using a continuous, solvent-free reactive extrusion setup that minimised extraction time and increase energy efficiencies compared to traditional batch processes. Although the overall yield of biochemicals in the liquor might seem low at first glance (maximum 7.5%), when the operating parameters are considered; that this extraction was achieved only through thermomechanical forces on untreated sawdust, without solvent, and with residence times of less than two minutes, this work shows that the continuous extraction of chemicals is possible with reactive extrusion. The obtained liquors are rich in hemicellulose-derived sugars, furans such as furfural and 5-HMF, and lignin compounds such as vanillin, and could be a source for valuable platform chemicals. For instance, furfural and their derivatives have been documented as effective treatments to get rid of fungi, insects, and nematodes, as fuel additives, wood modification agents, to name a few, indicating a huge market (Eseyin & Steele 2015). 5-HMF on the other hand is a platform chemical that can be used as petrochemical replacements in the chemical, plastic, food, and pharmaceutical industries (Kläusli 2014). The main application areas for vanillin are in food, beverage, and flavour industries, in addition to animal feeds, perfumes, cosmetics, and pharmaceutical industries (Olatunde et al. 2022).

Despite this proof-of-concept work demonstrating the viability of the process, further investigative work is required to fully realise the potential of reactive extrusion and to bring the technology to a market ready state. Possible future work would need to increase the yield and selectivity of potential products in order to allow isolation of sufficient quantities efficiently to make the process economically viable. There are several strategies and approaches that could achieve this. Firstly, the optimal extruder parameters, such as temperatures, screw design, screw speeds etc., requires further investigation in order to target certain products. Our work showed the production of biochemicals at 275 °C gave a volatile fraction rich in vanillin and coniferyl aldehyde that could be easily separated from the rest of the liquor via a simple solvent–solvent extraction. A screw design enabling higher levels of dissipated energy could potentially facilitate the depolymerisation of lignin into these valuable lignin monomers at this temperature. Similarly, such a harsher screw design would be required to fully break the highly crystalline cellulose domains to boost yields of glucose derived chemicals such as 5-HMF and levulinic acid. Another strategy is to modify the biomass. There was a conscious decision to not modify the sawdust aside from sieving to remove particles that would block the extruder, and the moisture content, since the study’s focus was on the viability of the process rather than the products. A pretreatment step of the biomass, either chemical, mechanical or biological, could be applied to boost yields or to target certain compounds (Joshi & Manjare 2024; Prasad et al. 2023). Another approach could be to co-extrude the biomass with catalysts or other additives, such as acids or bases, to assist with the breakdown of LCB biopolymers. Finally, the viability for other LCB sources such as grasses, leaves, bagasse, etc. would need to be studied to verify the variety of potential feedstocks.

## Conclusion

This work describes twin-screw extrusion technology as a continuous extraction process to produce biochemicals from sawdust for the first time. Moisture content was identified as the key parameter influencing the processability of wood particles during extrusion. A moisture content like undried timber (~ 55%) caused processability issues, indicating that some pre-drying of the wood to remove surface water is required before extrusion. The absence of kneading elements, which facilitate particle breakdown, led to blockages in the extrusion process. This highlights the potential advantage of twin-screw over single-screw or auger-based systems in reactive extrusion of biomass, due to its superior capacity for mechanical energy transfer and particle size reduction. In this study, screw speed had no significant impact on biochemical output. Although feed rate was not assessed in this research, the observed flexibility in screw speed suggests a level of process robustness that warrants further investigation. Extrusion temperature is another critical parameter influencing the breakdown of lignocellulosic biomass components. Processing at 275 °C resulted in mild lignin breakdown, while at 375 °C, approximately 20% of lignin was converted into degradation products. At this higher temperature, cellulose removal reached up to 18%, and hemicellulose removal was as high as 65%. The extracted liquors contained 4.5–7.5% chemicals, predominantly water-soluble oligomeric sugars and lignin–carbohydrate complexes. The oligomers were identified as originating from hemicellulose and represent a rich source of pentoses. Volatile chemicals recovered in considerable quantities included furfural, 5-HMF, vanillin, and coniferyl aldehyde.

Overall, the twin-screw reactive extrusion of sawdust offers a fast pathway for producing a liquor rich in biomass-derived chemicals. The advantages of using a solvent-free and continuous process could potentially overcome some of the problems of traditional biorefinery practices, making reactive extrusion a viable technology for future biorefinery development.

## Supplementary Information

Below is the link to the electronic supplementary material.

Supplementary Material 1

## Abbreviations

IEA: International energy agency
LCB: Lignocellulosic biomass
NaOH: Sodium hydroxide
L/D: Lengthdiameter ratio
D: Diameter
°C: Degree Celsius
rpm: Rotations per minute
kg/h: Kilogram per hour
h: Hour
g: Grams
w: Weight
ANOVA: Analysis of variance
p: Probability value
w/w: Weight by weight
MC: Moisture content
ASTM: American society for testing and materials
mV: Millivolt
GC-FID: Gas chromatography flame ionisation detection
µL: Microliter
ppm: Parts per million
TAPPI: Technical association of the pulp and paper industry
HPIC: High pressure ion chromatography
GC–MS: Gas chromatography mass spectroscopy
min: Minutes
NMR: Nuclear magnetic resonance
MHz: Megahertz
2D: two-Dimensional
HSQC: Heteronuclear single quantum coherence
Hz: Hertz
CDCl_3_: Chloroform
*d6*-DMSO: Dimethyl sulfoxide-d6
SI: Supplementary information
CP MAS: Cross polarisation magic angle spinning
KL: Klason lignin
ASL: Acid soluble lignin
Glc: Glucan
Ara: Arabinan
Xyl: Xylan
Gal: Galactan
Man: Mannan
VFA: Volatile fatty acid

## Declarations

### Data availability

The datasets used and/or analysed during the current study and not included in the Supporting Information are available from the corresponding author on reasonable request.
