# Supplementary material for: Thermomechanical biorefining of Pinus radiata biomass to produce biochemicals using reactive extrusion
Source: Bioresour Bioprocess. 2025 Nov 5;12(1):131. doi: 10.1186/s40643-025-00971-9 (PMC12589751; doi:10.1186/s40643-025-00971-9)
Supplement: Supplementary file 1 — Supplementary Material 1 [file 40643_2025_971_MOESM1_ESM.docx]

**Supporting Information**

**Thermomechanical biorefining of *Pinus radiata* biomass to produce biochemicals using reactive extrusion**

*Beatrix Theobald^‡^, Aaron Tay ^‡^, Sumanth Ranganathan, Queenie Tanjay, Sunita Patel, Rebecca van Leeuwen, Marc Gaugler**

*^‡^ - Both authors contributed equally*

*Scion Group, Bioeconomy Science Institute, Titokorangi Drive, Private Bag 3020, Rotorua 3046, New Zealand*

**Corresponding author:*

*Marc Gaugler,* [*Marc.gaugler@scionresearch.com*](mailto:Marc.gaugler@scionresearch.com)

**Table S1: Summary of Screw configuration A with screw element, function, and dimensions.**

**Table S2: Summary of Screw configuration B with screw element, function, and dimensions.**

**Table S3: ANOVA TWO-WAY with replication for screw speed and moisture content.**

**Figure S1: Representative spectrum of quantification of VFA content in the extrusion liquor by GC-FID.**

**Figure S2: Comparison of the GC-MS spectra of the volatile chemicals in the liquors obtained at 275, 325, and 375 °C.**

**Table S4: Structure of selected compounds identified in the GC-MS spectra of extrusion liquors.**

**Figure S3: ^13^C NMR spectrum of the water-soluble fraction of extrusion liquor obtained at 325 °C.**

**Figure S4: ^13^C NMR spectrum of the water-soluble fraction of extrusion liquor obtained at 375 °C.**

**Figure S5: 2D-HSQC NMR spectrum of the water-soluble fraction of extrusion liquor obtained at 275 °C.**

**Figure S6: 2D-HSQC NMR spectrum of the water-soluble fraction of extrusion liquor obtained at 375 °C.**

**Figure S7: ^1^H (a) and ^13^C (b) NMR spectra of the CDCl_3_ extracted liquor produced at 275 °C.**

**Figure S8: ^1^H (a) and ^13^C (b) NMR spectra of the CDCl_3_ extracted liquor produced at 325 °C.**

**Figure S9: ^1^H (a) and ^13^C (b) NMR spectra of the CDCl_3_ extracted liquor produced at 375 °C.**

**Table S5: Selected literature ^1^H and ^13^C NMR data for major identifiable compounds found in the CDCl_3_ extracted phase of the extrusion liquor.**

**Table S1. Summary of Screw configuration A with screw element, function, and dimensions.**

| **Screw element type** | **Element length** | | **Screw position** | |
| --- | --- | --- | --- | --- |
|  | **D unit** | **mm** | **D unit** | **mm** |
| conveying forward undercut - Single flights 26/26 | 1 | 26 | 1 | 26 |
| conveying forward undercut - Single flights 26/26 | 1 | 26 | 2 | 52 |
| conveying forward - double flights 26/26 | 1 | 26 | 3 | 78 |
| conveying forward - double flights 26/26 | 1 | 26 | 4 | 104 |
| conveying forward - double flights 26/26 | 1 | 26 | 5 | 130 |
| conveying forward - double flights 26/26 | 1 | 26 | 6 | 156 |
| conveying forward - double flights 26/26 | 1 | 26 | 7 | 182 |
| conveying forward - double flights 26/26 | 1 | 26 | 8 | 208 |
| conveying forward - double flights 26/26 | 1 | 26 | 9 | 234 |
| Spacer-ring | 1 | 26 | 10 | 260 |
| conveying forward - double flights 26/26 | 1 | 26 | 11 | 286 |
| conveying forward - double flights 26/26 | 1 | 26 | 12 | 312 |
| conveying forward - double flights 26/26 | 1 | 26 | 13 | 338 |
| Spacer-ring | 1 | 26 | 14 | 364 |
| conveying forward - double flights 26/26 | 1 | 26 | 15 | 390 |
| conveying forward - double flights 26/26 | 1 | 26 | 16 | 416 |
| conveying forward - double flights 26/26 | 1 | 26 | 17 | 442 |
| Spacer-ring | 1 | 26 | 18 | 468 |
| conveying forward - double flights 26/26 | 1 | 26 | 19 | 494 |
| conveying forward - double flights 26/26 | 1 | 26 | 20 | 520 |
| conveying forward - double flights 26/26 | 1 | 26 | 21 | 546 |
| Spacer-ring | 1 | 26 | 22 | 572 |
| conveying forward - double flights 26/26 | 1 | 26 | 23 | 598 |
| conveying forward - double flights 26/26 | 1 | 26 | 24 | 624 |
| conveying forward - double flights 26/26 | 1 | 26 | 25 | 650 |
| Spacer-ring | 1 | 26 | 26 | 676 |
| conveying forward - double flights 26/26 | 1 | 26 | 27 | 702 |
| conveying forward - double flights 26/26 | 1 | 26 | 28 | 728 |
| conveying forward - double flights 26/26 | 1 | 26 | 29 | 754 |
| conveying forward - double flights 26/26 | 1 | 26 | 30 | 780 |
| conveying forward - double flights 26/26 | 1 | 26 | 31 | 806 |
| conveying forward - double flights 26/26 | 1 | 26 | 32 | 832 |
| conveying forward - double flights 26/26 | 1 | 26 | 33 | 858 |
| conveying forward - double flights 26/26 | 1 | 26 | 34 | 884 |
| conveying forward - double flights 26/26 | 1 | 26 | 35 | 910 |
| conveying forward - double flights 26/26 | 1 | 26 | 36 | 936 |
| conveying forward - double flights 26/26 | 1 | 26 | 37 | 962 |
| conveying forward - double flights 26/26 | 1 | 26 | 38 | 988 |
| conveying forward - double flights 26/13 | 0.5 | 13 | 38.5 | 1001 |
| conveying discharge forward - double flights 13/39 | 1.5 | 39 | 40 | 1040 |

**Table S2. Summary of Screw configuration B with screw element, function, and dimensions.**

| **Screw element type** | **Element length** | | **Screw position** | |
| --- | --- | --- | --- | --- |
|  | **D unit** | **mm** | **D unit** | **mm** |
| conveying forward undercut - Single flights 26/26 | 1 | 26 | 1 | 26 |
| conveying forward undercut - Single flights 26/26 | 1 | 26 | 2 | 52 |
| conveying forward - double flights 26/26 | 1 | 26 | 3 | 78 |
| conveying forward - double flights 26/26 | 1 | 26 | 4 | 104 |
| conveying forward - double flights 26/26 | 1 | 26 | 5 | 130 |
| conveying forward - double flights 26/26 | 1 | 26 | 6 | 156 |
| conveying forward - double flights 26/26 | 1 | 26 | 7 | 182 |
| conveying forward - double flights 26/26 | 1 | 26 | 8 | 208 |
| conveying forward - double flights 26/26 | 1 | 26 | 9 | 234 |
| Individual Kneading Disc 0° angle | 0.25 | 6.5 | 9.25 | 240.5 |
| Individual Kneading Disc 90° angle | 0.25 | 6.5 | 9.5 | 247 |
| Individual Kneading Disc 0° angle | 0.25 | 6.5 | 9.75 | 253.5 |
| Individual Kneading Disc 90° angle | 0.25 | 6.5 | 10 | 260 |
| conveying forward - double flights 26/26 | 1 | 26 | 11 | 286 |
| conveying forward - double flights 26/26 | 1 | 26 | 12 | 312 |
| conveying forward - double flights 26/26 | 1 | 26 | 13 | 338 |
| Individual Kneading Disc 0° angle | 0.25 | 6.5 | 13.25 | 344.5 |
| Individual Kneading Disc 90° angle | 0.25 | 6.5 | 13.5 | 351 |
| Individual Kneading Disc 0° angle | 0.25 | 6.5 | 13.75 | 357.5 |
| Individual Kneading Disc 90° angle | 0.25 | 6.5 | 14 | 364 |
| conveying forward - double flights 26/26 | 1 | 26 | 15 | 390 |
| conveying forward - double flights 26/26 | 1 | 26 | 16 | 416 |
| conveying forward - double flights 26/26 | 1 | 26 | 17 | 442 |
| Individual Kneading Disc 0° angle | 0.25 | 6.5 | 17.25 | 448.5 |
| Individual Kneading Disc 90° angle | 0.25 | 6.5 | 17.5 | 455 |
| Individual Kneading Disc 0° angle | 0.25 | 6.5 | 17.75 | 461.5 |
| Individual Kneading Disc 90° angle | 0.25 | 6.5 | 18 | 468 |
| conveying forward - double flights 26/26 | 1 | 26 | 19 | 494 |
| conveying forward - double flights 26/26 | 1 | 26 | 20 | 520 |
| conveying forward - double flights 26/26 | 1 | 26 | 21 | 546 |
| Individual Kneading Disc 0° angle | 0.25 | 6.5 | 21.25 | 552.5 |
| Individual Kneading Disc 90° angle | 0.25 | 6.5 | 21.5 | 559 |
| Individual Kneading Disc 0° angle | 0.25 | 6.5 | 21.75 | 565.5 |
| Individual Kneading Disc 90° angle | 0.25 | 6.5 | 22 | 572 |
| conveying forward - double flights 26/26 | 1 | 26 | 23 | 598 |
| conveying forward - double flights 26/26 | 1 | 26 | 24 | 624 |
| conveying forward - double flights 26/26 | 1 | 26 | 25 | 650 |
| Individual Kneading Disc 0° angle | 0.25 | 6.5 | 25.25 | 656.5 |
| Individual Kneading Disc 90° angle | 0.25 | 6.5 | 25.5 | 663 |
| Individual Kneading Disc 0° angle | 0.25 | 6.5 | 25.75 | 669.5 |
| Individual Kneading Disc 90° angle | 0.25 | 6.5 | 26 | 676 |
| conveying forward - double flights 26/26 | 1 | 26 | 27 | 702 |
| conveying forward - double flights 26/26 | 1 | 26 | 28 | 728 |
| conveying forward - double flights 26/26 | 1 | 26 | 29 | 754 |
| Individual Kneading Disc 0° angle | 0.25 | 6.5 | 29.25 | 760.5 |
| Individual Kneading Disc 90° angle | 0.25 | 6.5 | 29.5 | 767 |
| Individual Kneading Disc 0° angle | 0.25 | 6.5 | 29.75 | 773.5 |
| Individual Kneading Disc 90° angle | 0.25 | 6.5 | 30 | 780 |
| conveying forward - double flights 26/26 | 1 | 26 | 31 | 806 |
| conveying forward - double flights 26/26 | 1 | 26 | 32 | 832 |
| conveying forward - double flights 26/26 | 1 | 26 | 33 | 858 |
| conveying forward - double flights 26/26 | 1 | 26 | 34 | 884 |
| conveying forward - double flights 26/26 | 1 | 26 | 35 | 910 |
| conveying forward - double flights 26/26 | 1 | 26 | 36 | 936 |
| conveying forward - double flights 26/26 | 1 | 26 | 37 | 962 |
| conveying forward - double flights 26/26 | 1 | 26 | 38 | 988 |
| conveying forward - double flights 26/13 | 0.5 | 13 | 38.5 | 1001 |
| conveying discharge forward - double flights 13/39 | 1.5 | 39 | 40 | 1040 |

**Table S3. ANOVA TWO-WAY with replication for screw speed and moisture content.**

| SUMMARY | 30 | 40 | 50 | Total |  |  |
| --- | --- | --- | --- | --- | --- | --- |
| *50* |  |  |  |  |  |  |
| Count | 2 | 2 | 2 | 6 |  |  |
| Sum | 2.98935 | 5.559828 | 9.281139 | 17.83032 |  |  |
| Average | 1.49467 | 2.779914 | 4.64057 | 2.97172 |  |  |
| Variance | 0.03805 | 0.116976 | 0.002699 | 2.032946 |  |  |
|  |  |  |  |  |  |  |
| *100* |  |  |  |  |  |  |
| Count | 2 | 2 | 2 | 6 |  |  |
| Sum | 3.64396 | 4.961152 | 9.107429 | 17.71255 |  |  |
| Average | 1.82198 | 2.480576 | 4.553714 | 2.952091 |  |  |
| Variance | 0.00350 | 0.170063 | 0.05628 | 1.671837 |  |  |
|  |  |  |  |  |  |  |
| *300* |  |  |  |  |  |  |
| Count | 2 | 2 | 2 | 6 |  |  |
| Sum | 2.64676 | 5.519319 | 9.051835 | 17.21792 |  |  |
| Average | 1.32338 | 2.75966 | 4.525918 | 2.869654 |  |  |
| Variance | 0.01663 | 0.210933 | 0.161636 | 2.136343 |  |  |
|  |  |  |  |  |  |  |
| *Total* |  |  |  |  |  |  |
| Count | 6 | 6 | 6 |  |  |  |
| Sum | 9.28008 | 16.0403 | 27.4404 |  |  |  |
| Average | 1.54668 | 2.673383 | 4.573401 |  |  |  |
| Variance | 0.06298 | 0.121981 | 0.046985 |  |  |  |
|  |  |  |  |  |  |  |
|  |  |  |  |  |  |  |
| ANOVA |  |  |  |  |  |  |
| *Source of Variation* | *SS* | *df* | *MS* | *F* | *P-value* | *F crit* |
| Sample | 0.03519 | 2 | 0.017599 | 0.203906 | 0.819204 | 4.256495 |
| Columns | 28.0811 | 2 | 14.04055 | 162.6793 | 8.61E-08 | 4.256495 |
| Interaction | 0.34775 | 4 | 0.086939 | 1.007312 | 0.452527 | 3.633089 |
| Within | 0.77677 | 9 | 0.086308 |  |  |  |
|  |  |  |  |  |  |  |
| Total | 29.2408 | 17 |  |  |  |  |


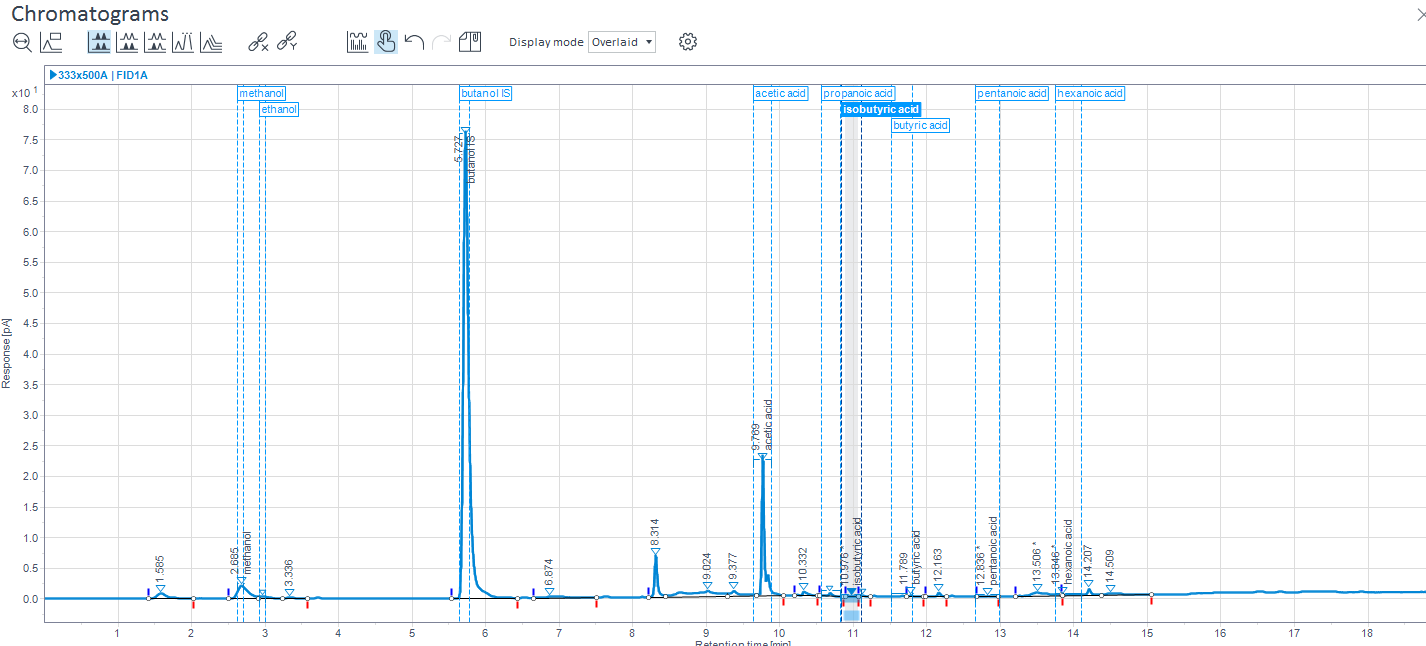


**Figure S1. Representative spectrum of quantification of VFA content in the extrusion liquor by GC-FID.**


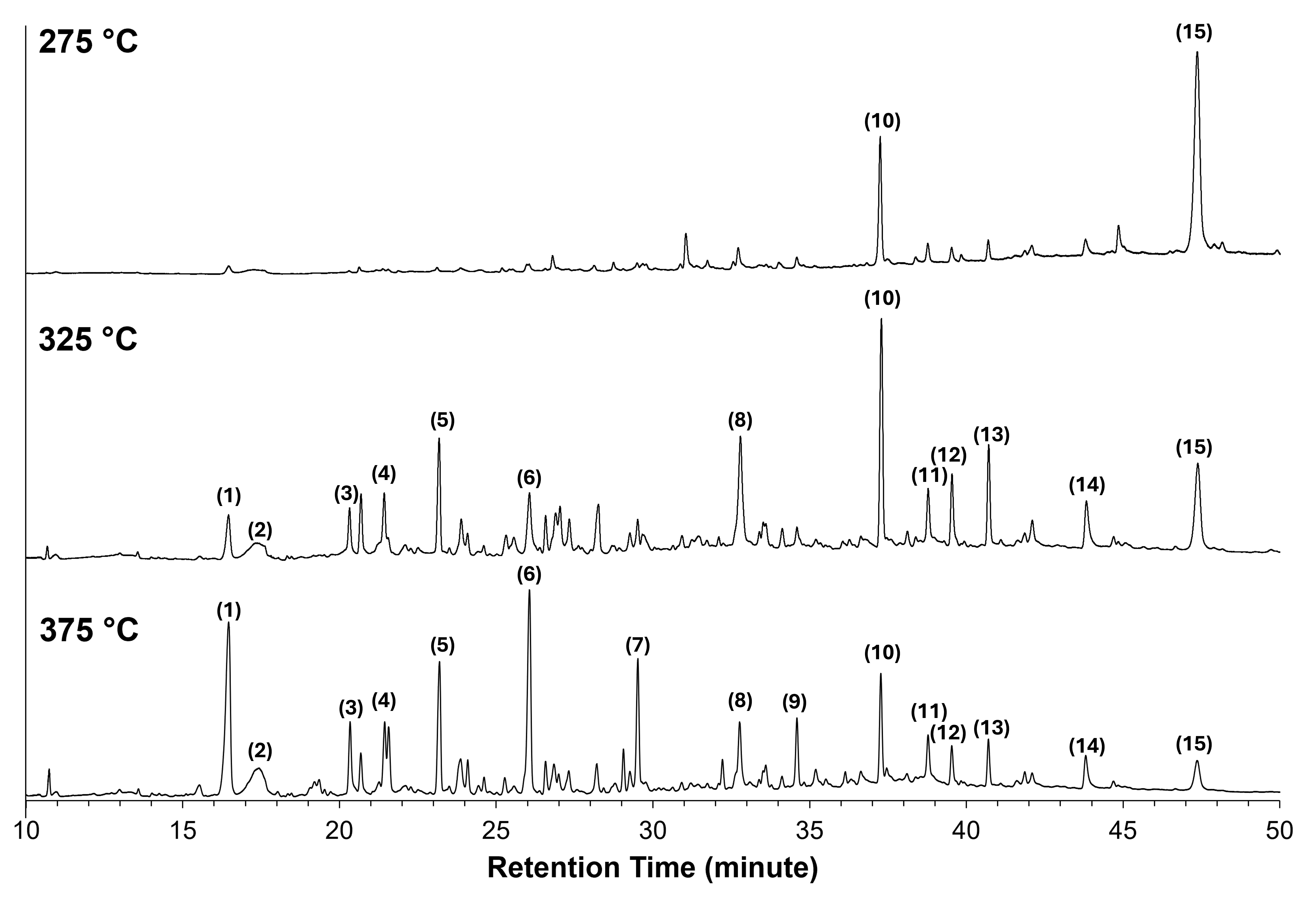


**Figure S2. Comparison of the GC-MS spectra of the volatile chemicals in the liquors obtained at 275, 325, and 375 °C.**

**Table S4. Structure of selected compounds identified in the GC-MS spectra of extrusion liquors.**

| **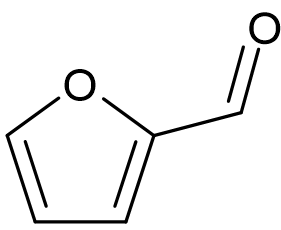Furfural**  **(1)** | **2-Furanmethanol**  **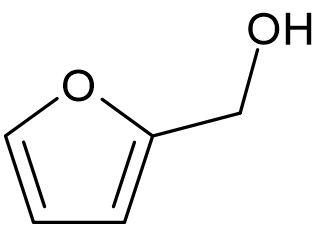**  **(2)** | **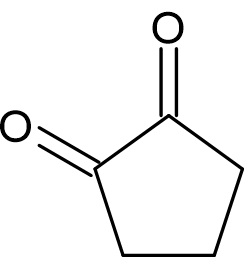1,2-Cyclopentadione**  **(3)** |
| --- | --- | --- |
| **2-(5H)-Furanone**  **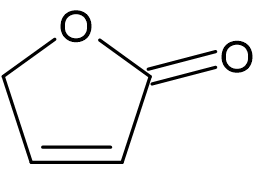**  **(4)** | **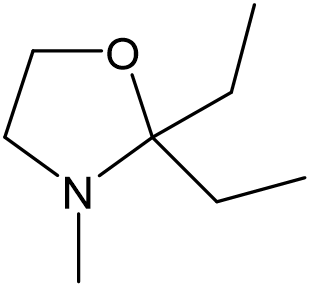Oxazolidine, 2,2-diethyl-3-methyl-**  **(5)** | **Guaiacol**  **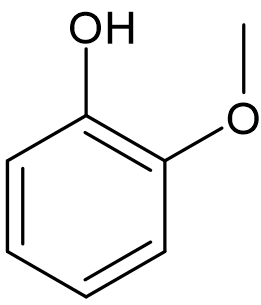**  **(6)** |
| **Creosol**  **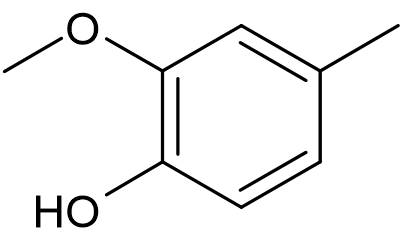**  **(7)** | **5-HMF**  **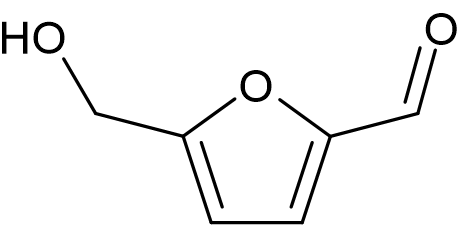**  **(8)** | **Eugenol**  **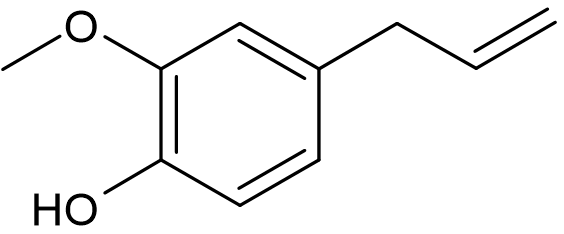**  **(9)** |
| **Vanillin**  **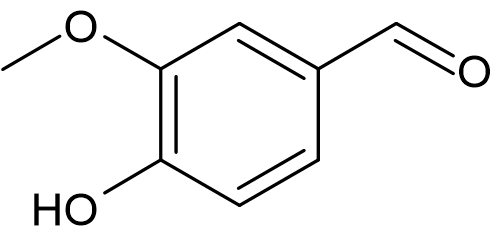**  **(10)** | **Phenol, 2-methoxy-4-propyl-**  **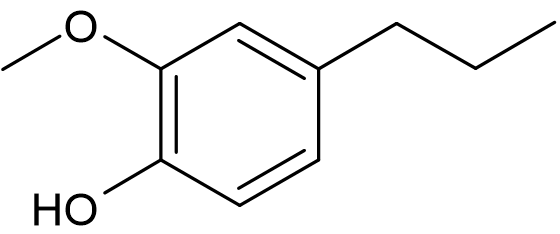**  **(11)** | **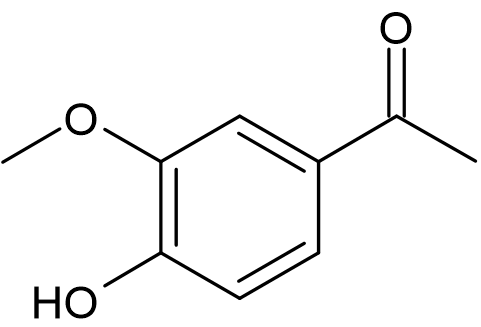Ethanone, 1-(3-hydroxy-4-methoxyphenyl)-**  **(12)** |
| **2-Propanone, 1-(4-hydroxy-3-methoxyphenyl)-**  **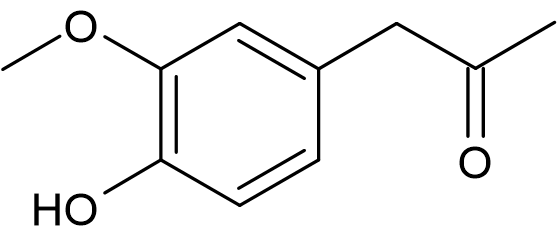**  **(13)** | **Benzenepropanol, 4-hydroxy-3-methoxy-**  **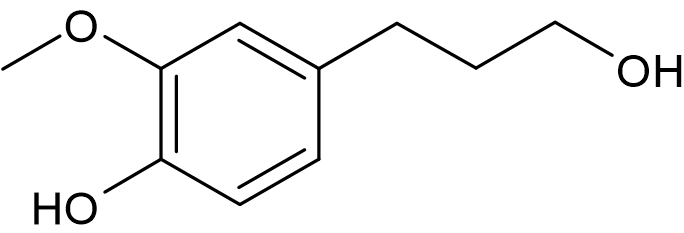**  **(14)** | **Coniferyl aldehyde**  **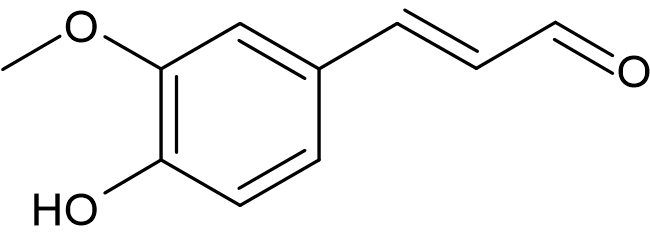**  **(15)** |


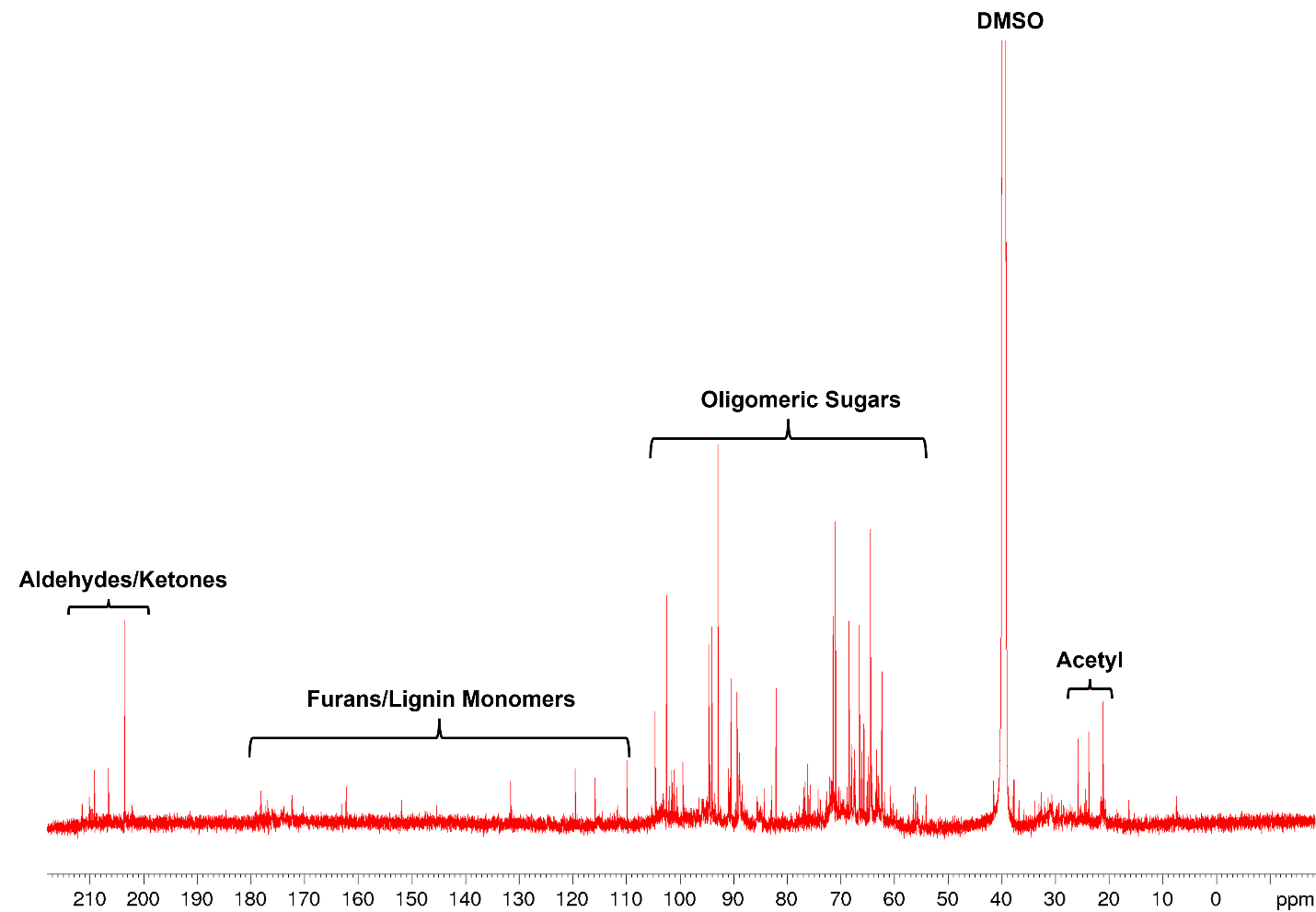


**Figure S3. ^13^C NMR spectrum of the water-soluble fraction of extrusion liquor obtained at 325 °C.**


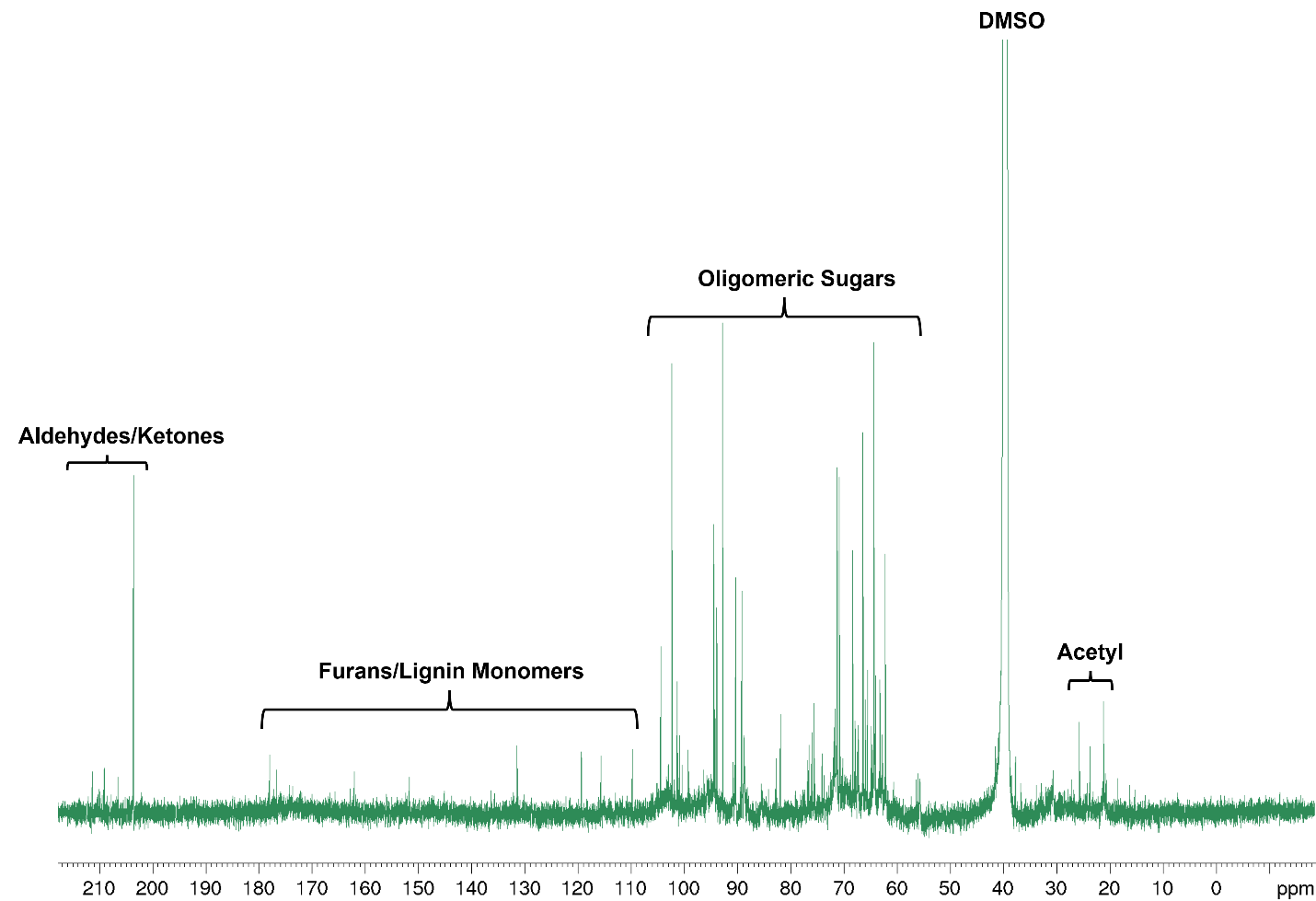


**Figure S4. ^13^C NMR spectrum of the water-soluble fraction of extrusion liquor obtained at 375 °C.**


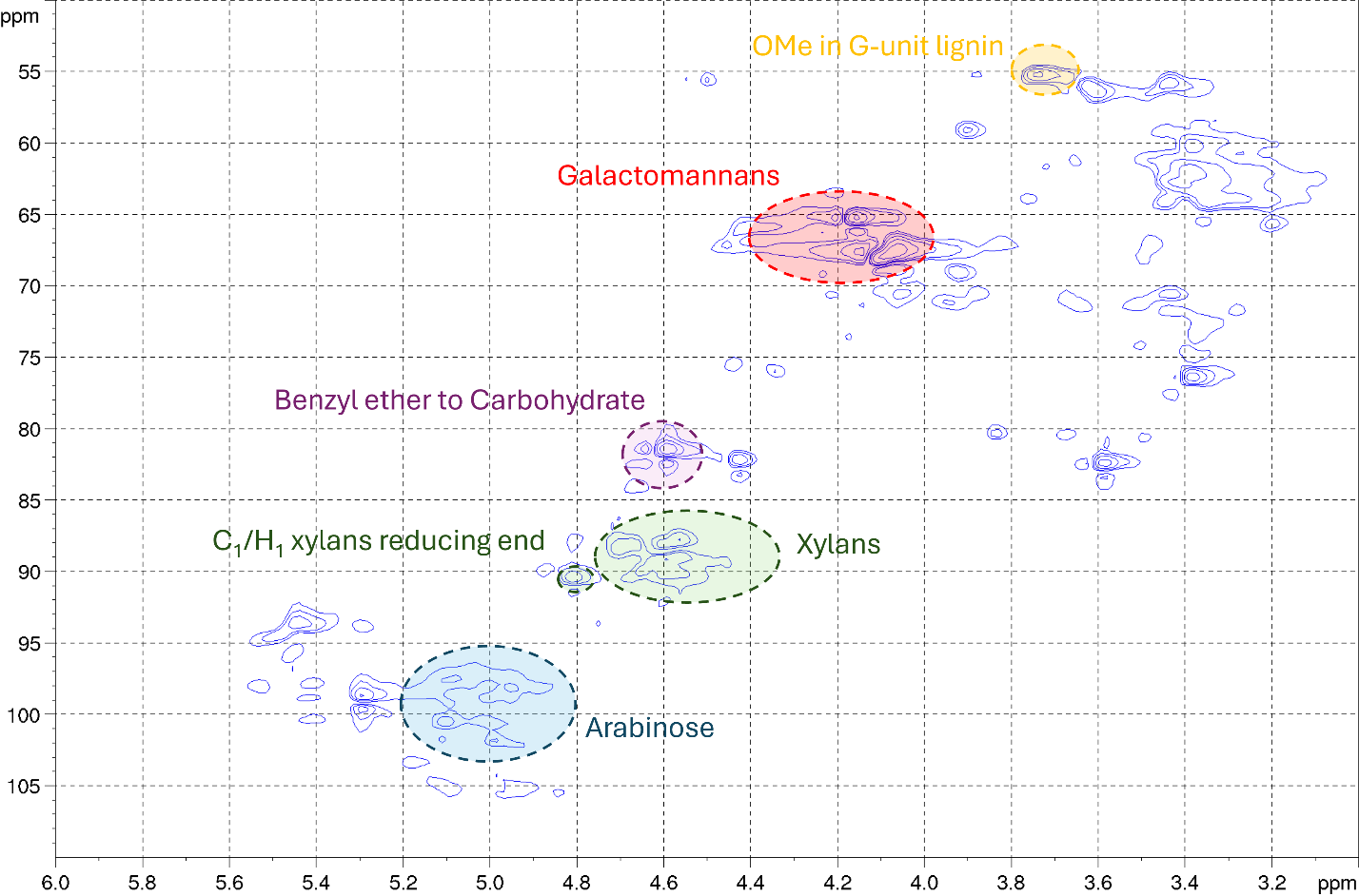


**Figure S5. 2D-HSQC NMR spectrum of the water-soluble fraction of extrusion liquor obtained at 275 °C.**


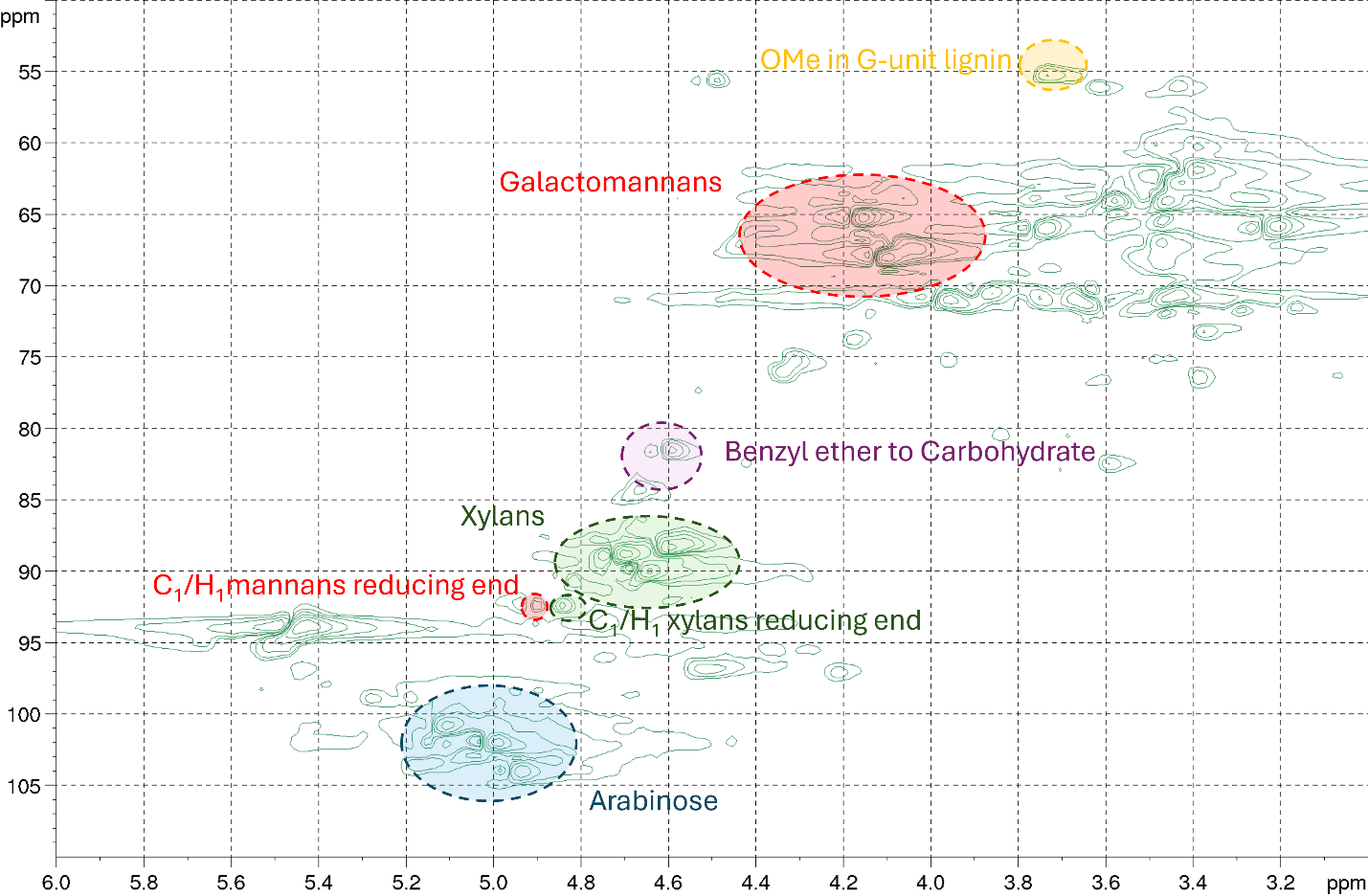


**Figure S6. 2D-HSQC NMR spectrum of the water-soluble fraction of extrusion liquor obtained at 375 °C.**


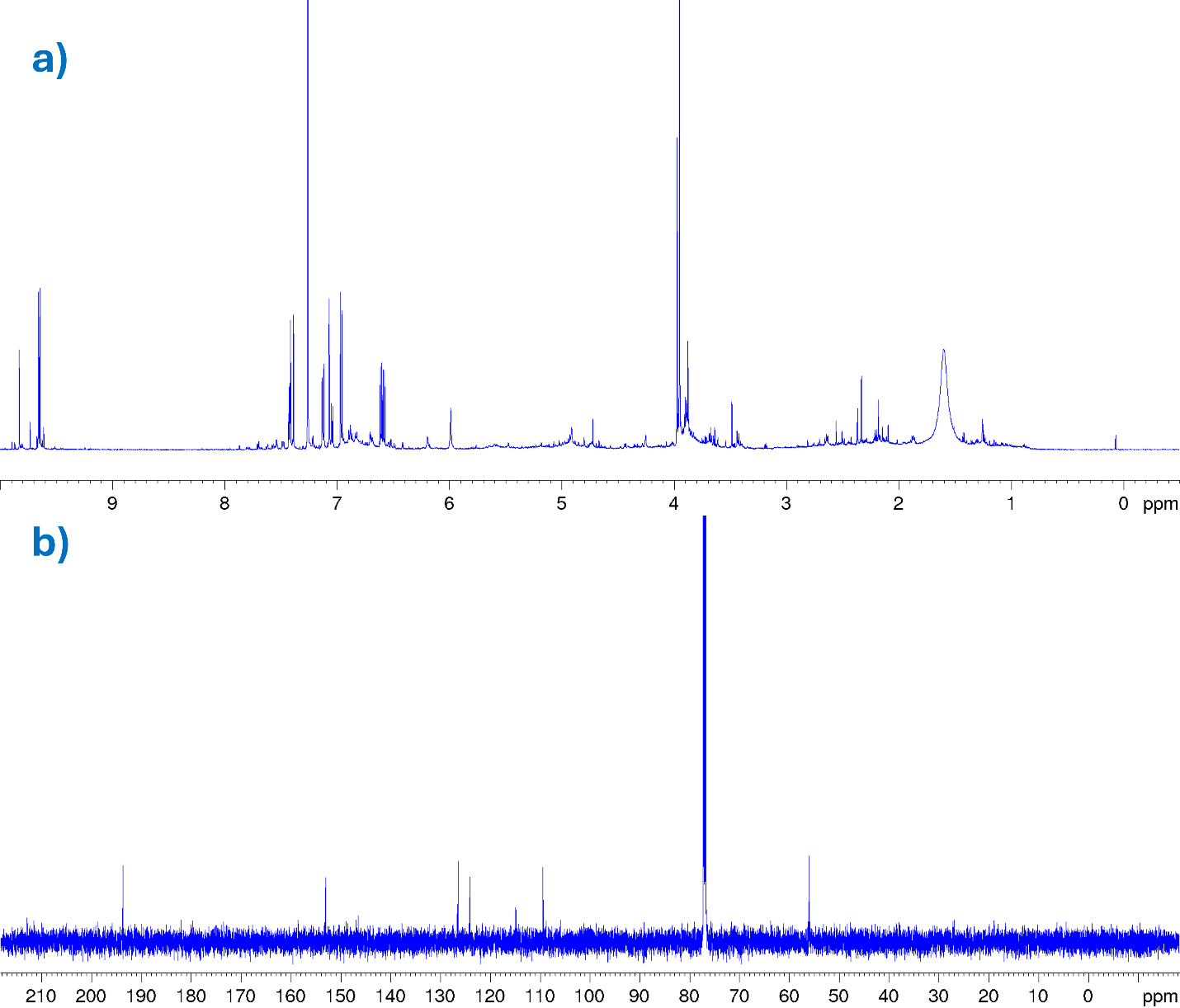


**Figure S7. ^1^H (a) and ^13^C (b) NMR spectra of the CDCl_3_ extracted liquor produced at 275 °C.**


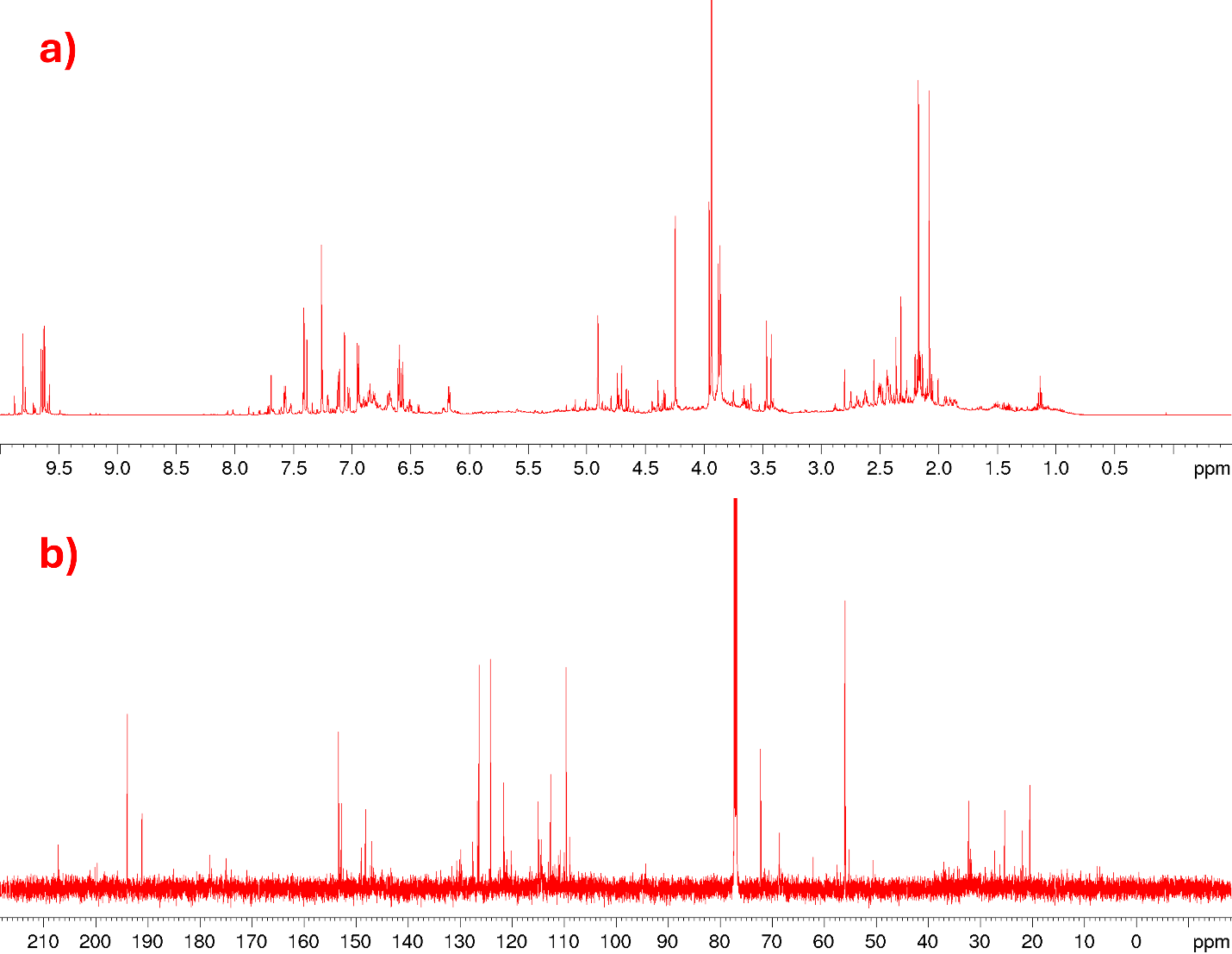


**Figure S8. ^1^H (a) and ^13^C (b) NMR spectra of the CDCl_3_ extracted liquor produced at 325 °C.**


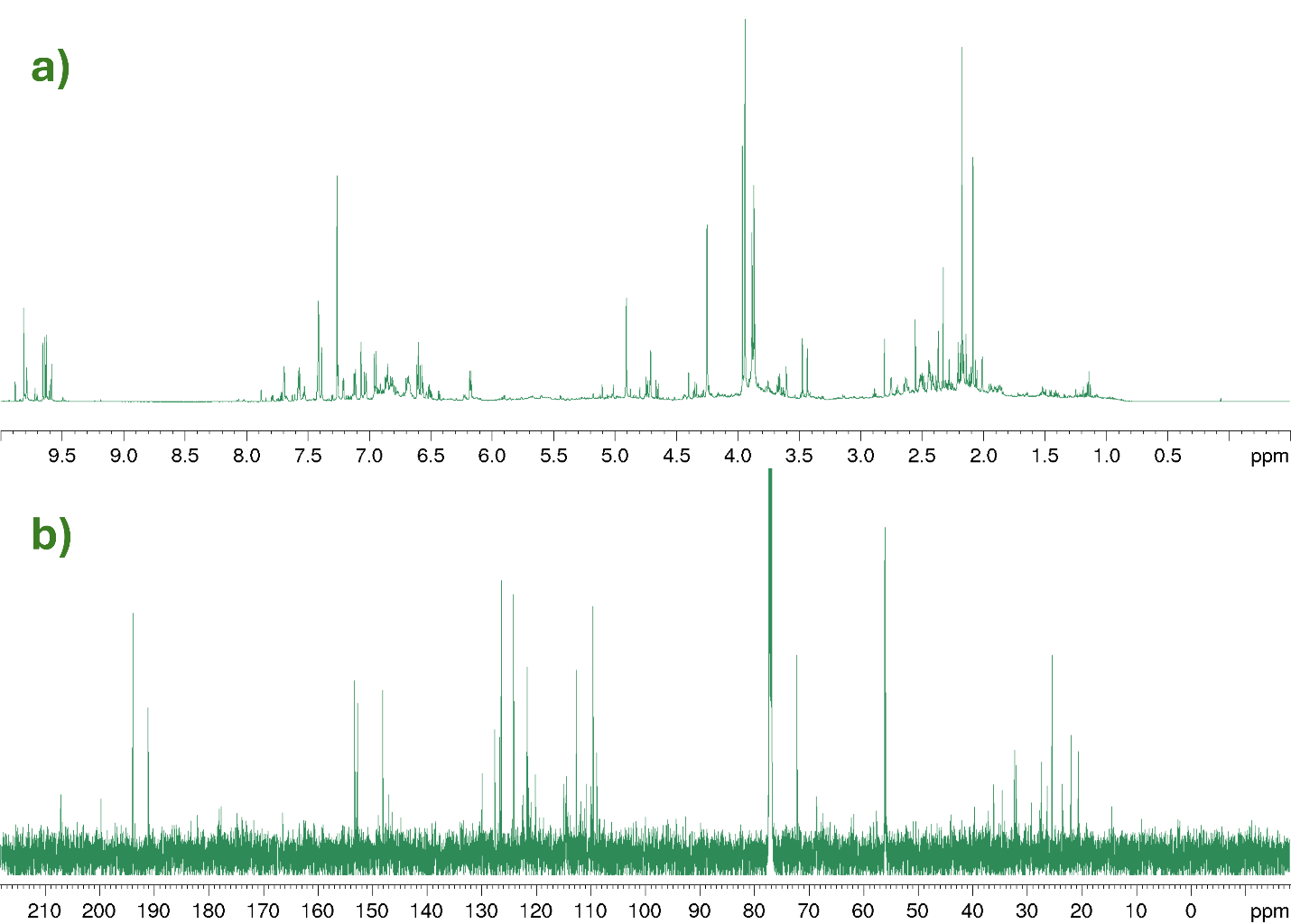


**Figure S9. ^1^H (a) and ^13^C (b) NMR spectra of the CDCl_3_ extracted liquor produced at 375 °C.**

**Table S5. Selected literature ^1^H and ^13^C NMR data for major identifiable compounds found in the CDCl_3_ extracted phase of the extrusion liquor.**

| **Compound** | **^1^H NMR Chemical shift** | **^13^C NMR Chemical shift** |
| --- | --- | --- |
| **Coniferyl aldehyde [1, 2]** | **3.95 (s, 3H), 5.97 (br s, 1H), 6.60 (dd, 1H), 6.96 (d, 1H), 7.07 (d, 1H), 7.13 (dd, 1H), 7.40 (d, 1H), 9.66 (d, 1H).** | **56.0, 109.5, 114.9, 123.9, 126.2, 126.5, 146.9, 148.9, 153.0, 193.4** |
| **Vanillin [2, 3]** | **3.98 (s, 3H), 6.15 (br s, 1H), 7.04 (d, 1H), 7.42 (d, 1H), 7.43 (dd, 1H), 9.83 (s, 1H)** | **56.1, 108.8, 114.4, 127.5, 129.9, 147.1, 151.7, 190.9** |
| **5-HMF [4]** | **3.51 (br s, 1H), 4.66 (s, 2H), 6.50 (d, 1H), 7.21 (d, 1H), 9.51 (s, 1H)** | **56.9, 110.1, 123.9, 151.8, 162.0, 178.1** |
| **Furfural [4]** | **6.58 (m, 1H), 7.24 (m, 1H), 7.67 (m, 1H), 9.64 (s, 1H)** | **112.5, 121.3, 148.1, 152.8, 177.8** |

**References**

1. Miyazawa, M. and M. Hisama, *Antimutagenic Activity of Phenylpropanoids from Clove (Syzygium aromaticum).* Journal of Agricultural and Food Chemistry, 2003. **51**(22): p. 6413-6422.

2. Hiltunen, E., T.T. Pakkanen, and L. Alvila, *Phenolic compounds in silver birch (Betula pendula Roth) wood.* 2006. **60**(5): p. 519-527.

3. Valente, C., et al., *Bioactive Diterpenoids, a New Jatrophane and Two ent-Abietanes, and Other Constituents from Euphorbia pubescens.* Journal of Natural Products, 2004. **67**(5): p. 902-904.

4. Mascal, M. and E.B. Nikitin, *Towards the Efficient, Total Glycan Utilization of Biomass.* ChemSusChem, 2009. **2**(5): p. 423-426.
